# Brucellosis and *Coxiella burnetii* Infection in Householders and Their Animals in Secure Villages in Herat Province, Afghanistan: A Cross-Sectional Study

**DOI:** 10.1371/journal.pntd.0004112

**Published:** 2015-10-20

**Authors:** Zarif Akbarian, Ghulam Ziay, Willy Schauwers, Bashir Noormal, Islam Saeed, Abul Hussain Qanee, Zabiullah Shahab, Tania Dennison, Ian Dohoo, Ronald Jackson

**Affiliations:** 1 Afghan National Public Health Institute, Ministry of Public Health, Kabul, Afghanistan; 2 Central Veterinary Diagnostic & Research Laboratory, Kabul, Afghanistan; 3 Animal Health Development Program, Kabul, Afghanistan; 4 General Directorate of Animal Health and Production, Ministry of Agriculture, Irrigation and Livestock, Kabul, Afghanistan; 5 University of Prince Edward Island, Charlottetown, Canada; 6 EpiCentre, Massey University, Palmerston North, New Zealand; University of Otago, NEW ZEALAND

## Abstract

**Background:**

Brucellosis and coxiellosis are known to be endemic in ruminant populations throughout Afghanistan, but information about their prevalence and factors that affect prevalence in householders and livestock under diverse husbandry systems and pastoral settings is sparse.

**Methods/Principal Findings:**

We conducted a cross-sectional survey to investigate the seroprevalence of brucellosis and *Coxiella burnetii* in humans and livestock in six secure districts in Herat from 26^th^ December 2012–17^th^ January 2013. A total of 204 households with livestock were surveyed in six Kuchi and five sedentary type villages. Blood samples from 1,017 humans, 1,143 sheep, 876 goats and 344 cattle were tested for brucellosis and Q fever. About one in six households (15.7%) had at least one *Brucella* seropositive person, about one in eight households (12.3%) had at least one *Brucella* seropositive animal and about one in four (24.5%) had either seropositive animals or humans. Ninety-seven percent of households had at least one *C*. *burnetii* seropositive person and 98.5% of households had one or more *C*. *burnetii* seropositive animals. Forty- seven householders had serological evidence of exposure to both *C*. *burnetii* and *Brucella* and eight animals were serologically positive for both diseases. Drinking unpasteurised milk (OR 1.6), treating animals for ticks (OR 1.4), milking sheep (OR 1.4), male gender (OR 1.4) and seropositivity to *Brucella* (OR 4.3) were identified as risk factors for seropositivity to *C*. *burnetii* in householders. Household factors associated with households having either *Brucella* seropositive animals or humans were Kuchi households (OR 2.5), having ≤4 rooms in the house (OR 2.9) and not owning land (OR 2.9).

**Conclusions:**

The results from this study provide baseline information for the planning and monitoring of future interventions against these diseases. The implementation of this study greatly improved collaboration, coordination and capability of veterinary and public health professionals from government, NGOs and donor funded projects.

## Author Summary

Our study alerted authorities to a hitherto unrecognised high prevalence of *C*. *burnetii* infections, acted as a catalyst for the introduction of a national vaccination programme for protection of sheep and goats from brucellosis using Rev1 vaccine, demonstrated the benefits of a coordinated approach and fostered a better understanding of the nature of infection in different hosts and of the constraints for control faced by government services. A notable feature of the study was the enthusiasm and interest displayed by all of the participants throughout, from heads of government services to field personnel and villagers. Livestock owners regard zoonoses as adversities that affect their livestock and members of their households and do not partition them separately as medical or veterinary problems. Control programs need to take that perception into account and wherever possible avoid vesting ownership separately into veterinary or public health agencies.

## Introduction

Brucellosis and coxiellosis are known to be endemic in ruminant populations throughout Afghanistan but information about their prevalence and factors that affect prevalence in householders and livestock under diverse husbandry systems and pastoral settings is sparse. Brucellosis in animals results in economic losses due to decreased productivity from abortions and reduced milk yield while the disease in humans can be severely debilitating, often with long- term adverse consequences for health [1]. *C*. *burnetii* causes abortions in domestic ruminants and fever, pneumonia, meningo-encephalitis and hepatitis in acute cases of Q fever in humans and endocarditis in chronic cases [2,3]. Little is known about the epidemiology of *C*. *burnetii* in animals and humans in Afghanistan although a United Nations Food and Agriculture (FAO) funded study found serological evidence of its occurrence in Bamyan province in 2011 [4]. Brucellosis and Q fever are likely to be severely under-diagnosed and reported as they are not specifically included in the list of diseases in the donor-funded Essential Package of Hospital Services and Basic Package of Health Services. Until the start of this study there were no facilities for testing for *C*. *burnetii* at the central veterinary and public health laboratories in Kabul or elsewhere in the Republic. It is highly likely that both *B*. *melitensis* and *B*. *abortus* are present in Afghanistan but there is currently no in-country diagnostic capability for their culture or differentiation.

The objectives of the study were to

- Determine the prevalence of seropositivity for brucellosis and *C*. *burnetii* in humans and livestock in 20 randomly selected households with livestock in each of 10 randomly selected secure villages in six of the 16 districts of Herat province;
- Identify factors associated with the risk of seropositivity to these diseases in households, members of households and their livestock;
- provide baseline information which may be used for design of control programs and evaluation of the efficacy of control program interventions.

## Methods

A cross-sectional survey to investigate the seroprevalence of seropositivity to brucellosis and *C*. *burnetii* in humans and livestock (cattle, sheep and goats) was conducted in six secure districts in Herat from 26^th^ December 2012–17^th^ January 2013. Secure villages were located in districts considered to be secure because of no overt signs of anti-government activities therein, thus enabling public health and veterinary government agencies and DCA teams to conduct routine activities safely. The population of interest was humans and female livestock (cattle, sheep and goats) of breeding age in households in selected villages in Herat Province. The principal epidemiologic unit of interest was households with consideration given to both animals and humans within each household. A total of 204 households were surveyed in 11 villages, six of which were Kuchi [5] and five were sedentary type. Sedentary villages were selected from a list provided by the Afghanistan Information Management System which listed all the villages in Afghanistan in 2006. Kuchi are nomadic or transhumant pastoralists. Their villages were selected from a list provided by the Dutch Committee for Afghanistan (DCA) and the veterinary field units supported by the DCA identified their locations as at October 2012. Up to date data on numbers of livestock in households were not available. The last livestock census, which was sample based, was conducted by FAO in 2002–2003. Villages were randomly selected using the Data Analysis Tool in Microsoft Excel 2007 and 20 plus five reserve households in each village were randomly selected from a list provided by village elders of all households with livestock. In each household blood samples were collected in sterile red top or serum separation vacutainers from up to five householders ≥9 and ≤60 years of age and up to 10 randomly selected female sheep and goats of breeding age and up to five female cattle of breeding age. The sample size of five householders was based on the average Afghan household size of 7.6 persons. Individual human data recorded at the time of sampling included age, sex, marital status, occupation and history of abortions (if married) and for animals were species, age and abortion history (see S1 File and S2 File in Supplementary Information). “Small ruminants” is used throughout the paper as the collective term for sheep and goats.

Data on Knowledge, Attitude and Practices (KAP) at the household level were recorded at an interview with a senior person in each household. The questionnaire contained 47 questions and about 250 variables for recording data on numbers of adult female livestock, whether they were milked, preparation, use and trading and purchase practices for dairy products, socio-economic data, medical histories and knowledge of brucellosis (see S3 File in Supplementary Information). All interviews were conducted and recorded in Dari and translated into English. The KAP data were entered into separate computers in Herat and Kabul by different data entry operators. Data validation was conducted by monitoring by supervisors during data collection in the field and again during translation into English. Cross checking of the separate data sets was performed with the Microsoft Excel compare tool. Unique village and household identifiers were used for linkage of data among the three data sets.

Sera were separated at the Provincial Veterinary Laboratory in Herat and stored in duplicate cryovials before transport to the Central Veterinary Diagnostic and Research Laboratory and the Central Public Health Laboratory in Kabul. Human sera were considered brucellosis positive if they were competitive ELISA test positive. Animal sera were considered brucellosis positive if they showed agglutination in the standard method Rose Bengal Test (RBT) and were competitive ELISA test positive. Competitive ELISA kits (COMPELISA) and *B*. *abortus* Rose Bengal Test (standard) antigen were procured from the Animal and Plant Health Agency, United Kingdom. Brucellosis competitive ELISA samples giving an optical density (OD) equal to or below 60% of the mean of the OD of the 4 conjugate control wells were considered positive. Human and animal sera were tested according to manufacturers’ instructions with *Coxiella burnetii* (Q fever) Phase I and Phase II IgG ELISAs (IBL International Hamburg, Germany) and the Q fever LSI ELISA kit (LSI, Lissieu, France) respectively. The IBL human sera results were recorded as Units and the LSI animal ELISA results were expressed as optical density Sample/Positive control ratios corrected for the negative control. Human sera were considered positive if the result expressed as units was ≥11 and animal sera were considered to be positive if the S/P ratio was ≥40.

### Data analysis

Estimates of prevalence (at the person and animal level) were calculated using robust standard errors (variance linearization) to account for clustering at the village and household levels. Variance linearization (also known as robust standard errors clustered on village) was used to estimate the variance of all prevalences and hence to derive the confidence intervals (CI). Information about the total sample size of the population at risk at each of the levels (village, household and individual) was not recorded so neither sampling weights nor finite population correction factors were applied in the analyses.

Multivariable models were built to evaluate factors that influenced seropositivity to *Brucella* and *C*. *burnetii* in humans, livestock and households. Data which could conceivably be associated with disease status were first examined with descriptive statistics and tested for associations between the outcome variables as a first screening process and variables significant at *p* < 0.10 were entered into the multivariate models. Multivariable mixed logistic regression models were built with random effects for village and household (except for the household-level analyses which just included a random effect for village). In some models, there was zero village-level variance once the household random effect was incorporated and in these cases the village-level random effect was dropped. Models were built using a manual backwards elimination process. Normality and heteroscedasticity of all random effects were evaluated graphically and unless noted, were considered acceptable. Statistical analyses were performed using Epi Info version 7.1.2.0, and Stata 13 (StataCorp LP). Graphics were constructed in Excel (Microsoft).

### Ethics statement

The study was approved by the Islamic Republic of Afghanistan Department of Public Health’s Institutional Review Board (Approval numbers 221665 and 221769). Study participants signed a consent form agreeing to participate prior to the survey and informed signed consent was obtained from all adult study participants and parents of children <18 years old at the time of sampling. Livestock owners provided verbal consent for sampling of their animals. Ethics approval for sampling of the animals for this study was not required for taking blood because it is a normal husbandry procedure and was supervised or conducted by veterinarians. This is in line with Animal Welfare legislation in New Zealand and current practice in the Ministry of Agriculture in Afghanistan.

## Results

### Human results

#### *Brucella* serology in humans

**Fig 1:**
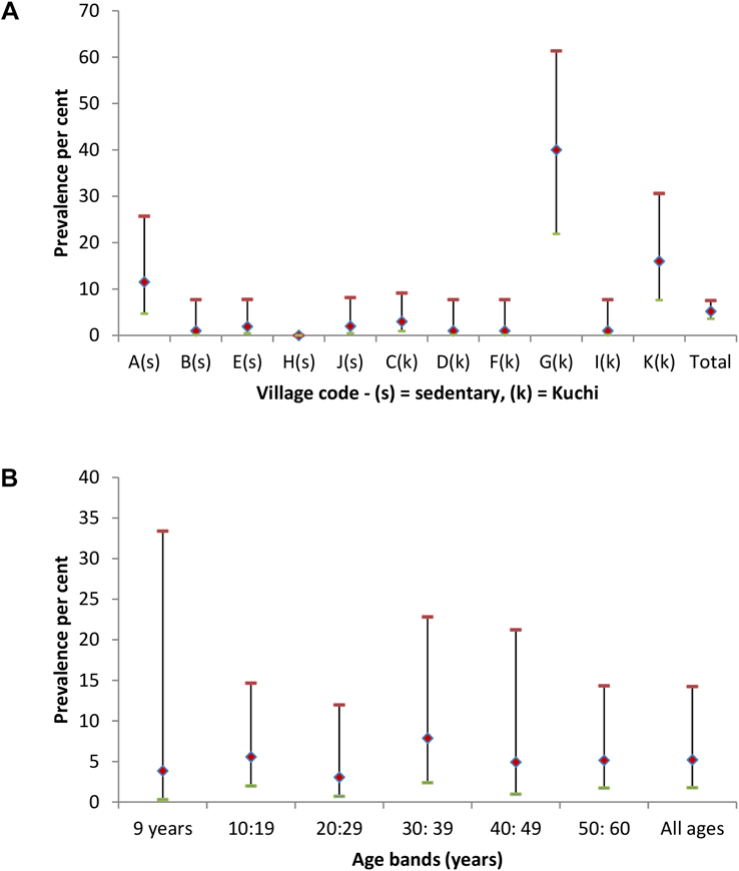
Human brucella seroprevalences (♦) with upper (-) and lower (-) 95% CI for each study village (A–K) in Fig 1A and categorised by age bands in Fig 1B. Sedentary villages in Fig 1A are identified by (s) and Kuchi by (k).

The overall prevalence of seropositives in the 1,017 humans tested in the 11 study villages was 5.2% (95% CI 1.8, 14.3) and seropositives were found in 10 villages. Humans with serological evidence of exposure to brucellosis were detected in 32 (15.7%) of the 204 study households and were strongly clustered in 3 villages (A, G and K); see Fig 1A. The prevalence of *Brucella* seropositives was slightly lower in females (4.1%, 95% CI 1.1, 13.8) than in males (7.2%, 95% CI 2.9, 16.5) and higher in Kuchi householders (7.1%, 95% CI 1.2, 32.5) than in sedentary village householders (3.3%, 95% CI 0.5, 17.7). Prevalences were similar across age bands (see Fig 1B). Of the 53 *Brucella* seropositive humans, 47 were also seropositive for *C*. *burnetii*.

#### *C*. *burnetii* serology in humans

**Fig 2:**
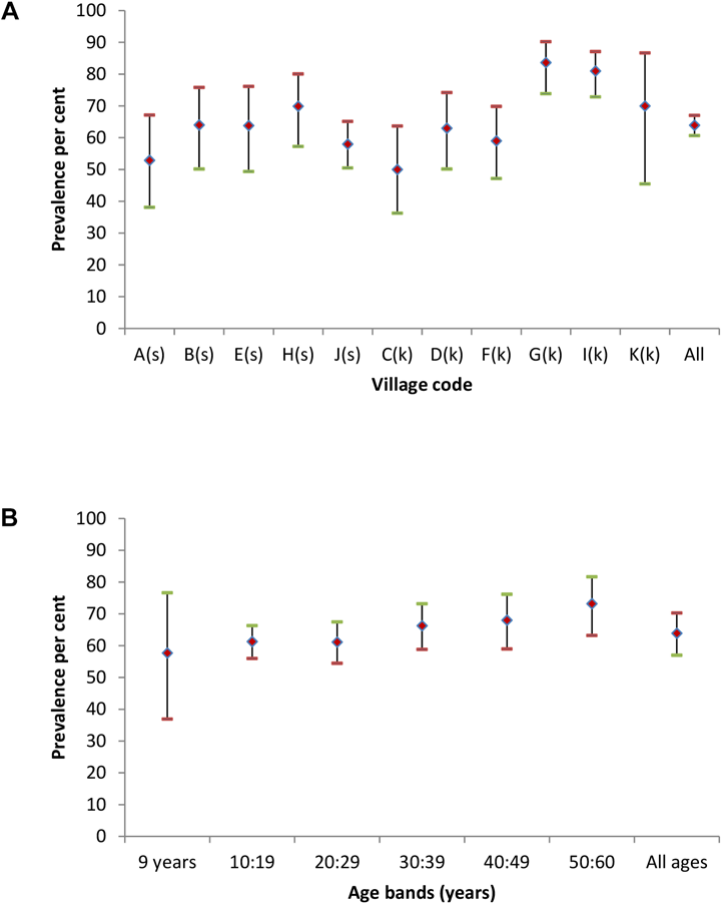
Human *C*. *burnetii* seroprevalences (♦) with upper (-) and lower (-) 95% CI for each study village (A–K) in Fig 2A and categorised by age bands in Fig 2B. Sedentary villages in Fig 2A are identified by (s) and Kuchi by (k).

The overall *C*. *burnetii* seroprevalence in humans in the 11 study villages was 63.9% (95% CI 57.0, 70.3) and seropositives were found in all villages. Humans with serological evidence of exposure to *C*. *burnetii* were detected in 199 (97.5%) of the 204 study households. The prevalence was slightly higher in males (69.3%, 95% CI 61.1, 76.5) than in females (61.0%, 95% CI 57.0, 64.7). Village level prevalences were similar (Fig 2A) but overall prevalences were slightly higher in Kuchi (66.1%, 95% CI 51.0, 78.6) than in sedentary villages (61.7%, 95% CI 53.3, 69.5). *C*. *burnetii s*eroprevalences categorised by 10 year age bands are shown in Fig 2B. Seroprevalence increased with increasing age from an initial high prevalence in the 26 persons, all aged 9 years, in the youngest age group.

#### Abortions and serological evidence of exposure to *C*. *burnetii* or *Brucella*

Miscarriages or abortions were reported by 146 (35.3%, 95% CI 28.7, 42.4) of the 414 married women for whom ages were recorded. Eight (5.5%, 95%CI 1.1, 23.7) of the women who reported that they had experienced an abortion were *Brucella* test positive and 101 (69.2%, 95%CI 58.0, 78.5) were *C*. *burnetii* test positive. Of the 414 married women of known age, the percentages of women reporting having had an abortion in three of the *Brucella* (B) by *C*. *burnetii* (C) categories were B negative C negative 28.8%, B negative C positive 38.3%, B positive C positive 57.1%. There was only one woman who was C negative and B positive.

### Animal results

#### *Brucella* serology in animals

Sera were separated from 1,143 blood samples from sheep, 876 from goats and 344 from cattle. There were 33 RBT positives of which 30 were ELISA positive. The seroprevalence was 1.4% (95% CI 0.7, 2.7) for sheep, 1.5% (95% CI 0.7, 3.0) for goats, 0.3% (95% CI 0.0, 3.2) for cattle and 1.3% (95% CI 0.7, 2.2) overall.

**Fig 3:**
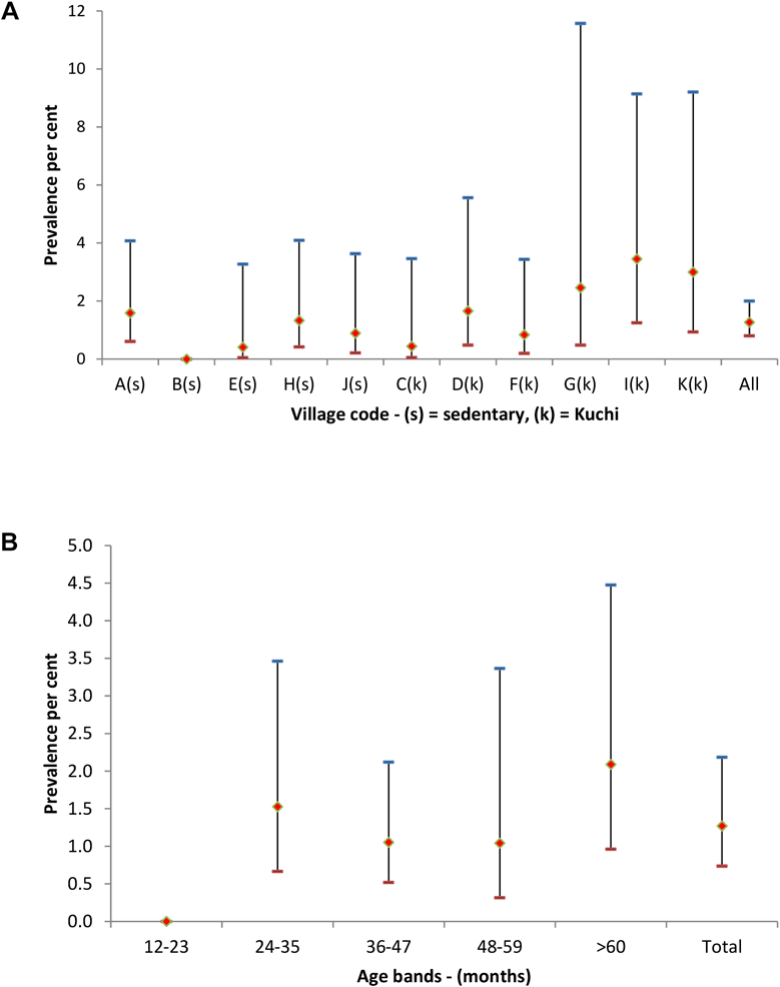
Animal brucella seroprevalences (♦) with upper (-) and lower (-) 95% ci for each study village (a–k) in fig 3a and categorised by age bands in fig 3b. Sedentary villages in fig 3a are identified by (s) and kuchi by (k).

*Brucella* seropositive animals were detected in 25 (12.3%) of the 204 study households and 10 of the 11 study villages. The prevalence in Kuchi villages was 1.8% (95% CI 0.8, 3.7) and 0.8% (95% CI 0.3, 2.3) in sedentary villages. The distribution of the prevalences of *Brucella* seropositive animals in the 11 study villages is shown in Fig 3A. There were no seropositive animals in the 12–23 month age group and the prevalence was relatively stable across the older age groups (Fig 3B).

#### *C*. *burnetii* serology in animals

**Fig 4:**
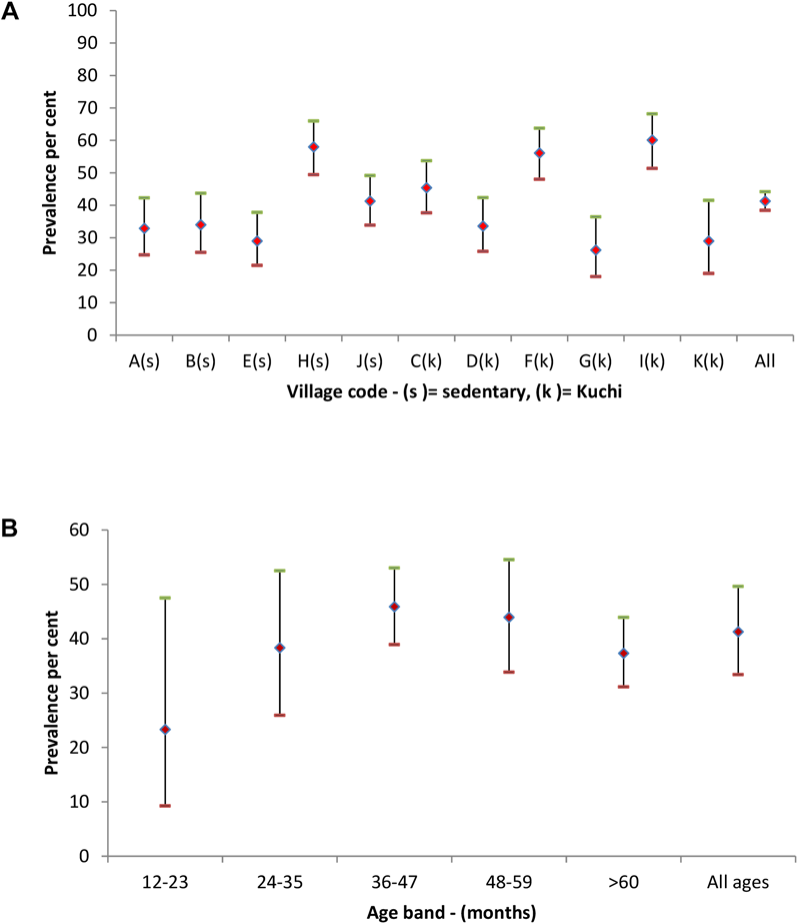
Animal *C*. *burnetii* seroprevalences (♦) with upper (-) and lower (-) 95% ci for each study village (a–k) in fig 4a and categorised by age bands in fig 4b. Sedentary villages in fig 4a are identified by (s) and kuchi by (k).

The prevalence of *C*. *burnetii* test positives in animals was 43.4% (95% CI 34.7, 52.5) for sheep, 52.7% (95% CI 43.8, 61.5) for goats, 5.2% (95% CI 3.1, 8.6) for cattle and 41.3% (95% CI 33.4, 49.6) overall. *C*. *burnetii* test positive animals were detected in all of the study villages and in 201 (98.5%) of the 204 study households. The respective prevalences for Kuchi and sedentary village animals were 44.3% (95% CI 31.0, 58.5) and 38.5% (95% CI 26.2, 52.6). Village level *C*. *burnetii* seroprevalences in animals are shown in Fig 4A. Ten goats and 10 sheep were both *Brucella* and *C*. *burnetii* seropositive. Age related seroprevalences showed an increase from the 12–23 month age band to the 36–49 month band after which the prevalence was reasonably stable (see Fig 4B).

### Multivariable models

#### *Brucella* seropositivity in animals

The mixed-effects logistic regression models of significant explanatory variables for *Brucella* seropositivity in small ruminants with households incorporated as a random effect are shown in Table 1. The effect of Kuchi was primarily evident in sheep where the OR was 5.3 if sheep and goats were separated out. The limited number of seropositive observations (n = 30), combined with the relatively small number of animals per household (N~ = 10) precluded a reliable evaluation of residuals at the household level (there was no evident variation at the village level). However, no obvious violations of assumptions were noted.

**Table 1:** Mixed-effects logistic regression results for *Brucella* seropositivity in small ruminants with households incorporated as a random effect.

Variables	Number	OR (95% CI)	P
**Kuchi village**	1,000	2.6 (1.1, 6.2)	0.03
**Sedentary village**	1,019	reference	
**Age**	continuous	1.03 (1.0, 1.1)	

Household effect variance 0.9 (0.2, 4.6)

#### *C*. *burnetii* seropositivity in animals

Only age was significant in the mixed effects logistic regression models of significant explanatory variables for *C*. *burnetii* seropositivity in small ruminants, sheep and goats with households and villages incorporated as random effects. Village type was not significant.

#### Abortions in animals

The numbers and incidence of abortions reported for each species and overall are shown in Table 2. The limited number of abortions (n = 184), combined with the relatively small number of animals per household (N~ = 10) precluded a reliable evaluation of residuals at the household level (there was no evident variation at the village level). However, no obvious violations of assumptions were noted.

**Table 2:** Numbers of abortions, numbers of study animals and incidence of abortions with 95% confidence intervals in brackets.

Species	N abortions (a)	N animals	Incidence% (95% CI)
**Sheep**	90	1,143	7.8 (5.9, 10.2)
**Goats**	87	876	9.9 (7.3, 13.4)
**Cattle**	7	344	2.0 (0.9, 4.3)
**All species**	184	2,363	(5.9, 10.2)

(a) refers to any occurrence during the animal’s life

#### Risk of abortion in animals

*Brucella* seropositivity and village type were associated with risk of abortions in small ruminants (see Table 3) but there was no association with *C*. *burnetii* seropositivity. Separate models for sheep and goats showed the risk of abortion was similar for sheep (OR 5.7, 95%CI 1.1, 29.2) and goats (OR 6.4, 95%CI 2.3, 17.6) and for village type. Households that reported abortions in their animals (n = 96) were more likely to have seropositive animals or humans (OR = 2.2 (95%CI 1.2, 4.2) than households which did not report abortions (n = 108).

**Table 3:** Mixed effects logistic regression results for risk of abortion in *Brucella* seropositive small ruminants with households incorporated as a random effect.

Variable	Number	OR (95% CI)	P
**Brucella seropositive**	29	6.4 (2,3, 17.6)	<0.01
**Brucella seronegative**	1,990	reference	
**Kuchi village**	1,000	0.6 (0.4, 1.1)	0.11
**Sedentary village**	1,019	reference	

Household effect variance 1.8 (1.1, 2.8)

### *Brucella* and *C*. *burnetii* seropositivity in householders

#### *Brucella* seropositivity in householders

Multivariable modelling with village and household incorporated as random effects showed that *C*. *burnetii* seropositivity was the only significant predictor (OR = 4.3, P = 0.003) for *Brucella* seropositivity in householders (village and household variance estimates 2.46 and 1.21 respectively)and *vice versa* for *Brucella* seropositivity as a predictor for *C*. *burnetii* seropositivity. As with the animal level data, there was limited ability to evaluate the distribution of residuals at the household level. Although no factors which could explain the variation in prevalence observed in Fig 1A, fitting a null model with village as the random effect confirmed that the variation in village prevalences was statistically significant. *C*. *burnetii* seropositivity in householders

Significant explanatory variables determined by multivariable modelling for *C*. *burnetii* seropositivity in householders with village and household incorporated as random effects are shown in Table 4.

**Table 4:** Mixed-effects logistic regression results for *C*. *burnetii* seropositivity in householders with households and villages incorporated as random effects.

Variables	Number	OR (95% CI)	P
**Males**	354	1.4 (0.96, 2.0)	0.06
**Females**	646	reference	
**Milk sheep**	564	1.4 (1.0, 2.0)	0.04
**Do not milk sheep**	436	reference	
**Drink raw milk**	326	1.6 (1.1, 2.3)	<0.01
**Do not drink raw milk**	624	reference	
**Treat animals for ticks**	609	1.4 (0.99, 1.9)	0.06
**Do not treat animals for ticks**	391	reference	
**Brucella test positive**	53	4.3 (1.6, 11.4)	<0.01
**Brucella test negative**	957	reference	

Household effect variance 0.6 (0.3, 1.2), Village effect variance 0.1 (0.02, 0.5)

### Knowledge, attitude and practices

#### Household disease exposure

The analysis for associations between household disease exposure status was confined to brucellosis and not conducted for Q fever because 199 of the 204 households had evidence of exposure to *C*. *burnetii* in their household members and 201 had evidence in their animals.

**Fig 5:**
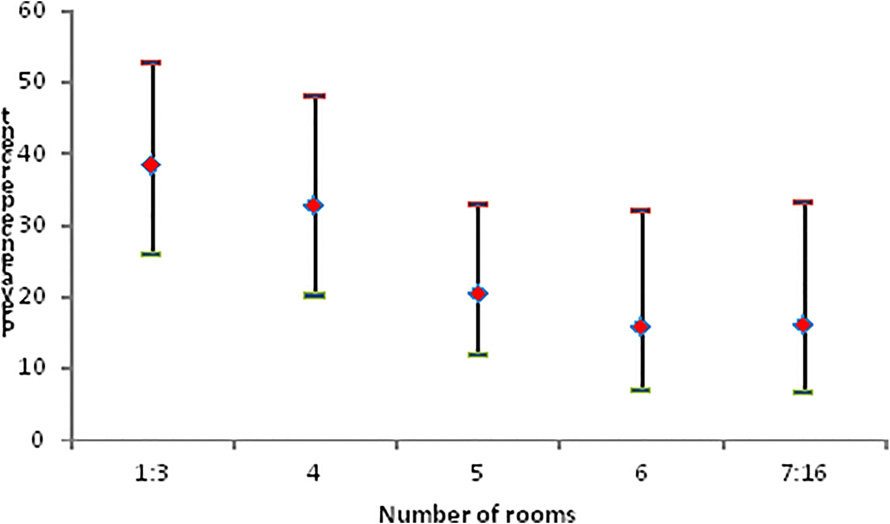
Point estimates (♦) and upper (-) and lower (-) 95% CI of prevalence of households with evidence of exposure to *Brucella* in animals or humans categorised by the number of rooms in the house.

There were 32 (15.7%) households with serological evidence of exposure to *Brucella* in humans, 25 (12.3%) with evidence in animals, 25 with evidence in householders, 50 (24.5%) with evidence for animals or humans and 7 (3.4%) for both animals and humans. The prevalence of households with serological evidence of exposure to *Brucella* in animals or humans categorised by number of rooms in households shows a downwards trend with increasing numbers of rooms (Fig 5).

The multivariable logistic regression model with households with serological evidence of exposure to *Brucella* in members of their household or in their animals as the outcome variable is shown in Table 5.

**Table 5:** Logistic regression results for risk of households having either *Brucella* seropositive animals or humans.

Variables	Number	OR (95% CI)	P
**Kuchi village household**	101	2.5 (0.9, 6.6)	0.07
**Sedentary village household**	103	reference	
**≤4 rooms in house**	87	2.9 (1.4, 6.1)	<0.01
**≥5 rooms in house**	117	reference	
**Family not owning land**	59	2.9 (1.3, 6.5)	0.01
**Family owning land**	145	reference	

Village effect variance 0.3 (0.03, 2.5)

#### Prevalence of occupational risks for *Brucella* and *C*. *burnetii*

The prevalence of householder involvements with husbandry of animals and preparation of animal products which could entail risk of exposure to *Brucella* and *C*. *burnetii* are shown in Table 6. Most householders were engaged in multiple activities with their animals; 29.8% with ≤5, 44% with 6 to 10 and 26.2% with 11 to 16 activities. These risk factors did not have a statistically significant association with either *Brucella* or *Coxiella* in our analyses, but this does not imply that they are not potentially important. Rather, it indicates that there was insufficient power in our study to detect such relationships.

**Table 6:** Prevalence of occupational risks for exposure to *Brucella* and *C*. *burnetii* among householders.

Occupational risks	Prevalence %
**Milk cow**	45.8
**Milk sheep**	56.5
**Milk goat**	53.8
**Prepare cheese**	23.5
**Prepare butter**	53.7
**Prepare yoghurt**	59.3
**Prepare other milk products**	15.8
**Slaughter animals**	19.8
**Feed animals**	82.9
**Clean animal barns**	86.4
**Assist animal births**	42.3
**Look after animals on pasture**	35.7
**Shear animals**	58.2
**Remove ticks from animals**	61.1
**Treat animals for ticks**	46.3
**Use un-boiled milk**	32.6

Washing hands with soap and water after handling animals at birthing was practised by 73% of households but only 33% reported burying or burning afterbirths or aborted foetuses. Most were disposed of by feeding to dogs or in the open environment.

## Discussion

Despite its conceptual appeal [6] and successful application over the past 20 years for infectious diseases such as bovine spongiform encephalopathy, highly pathogenic H5N1 avian influenza, *C*. *burnetii* and leptospirosis, linked public health and veterinary field investigations of important zoonoses for which the reservoir hosts are farmed ruminants have been rarely conducted in low income countries. Serological studies involving humans and their livestock have been reported for brucellosis in Kyrgyzstan [7], Mongolia [8] and Egypt [9], for *C*. *burnetii* in Egypt [10] and for both brucellosis and Q fever in Chad [11] and the authors are aware of several unpublished investigations for brucellosis in Central Asia and China. Our study was made possible by the terms of reference for the series of in-country studies in South Asia conducted under the Massey University European Commission funded and World Bank administered South Asia project which embodied veterinary and public health collaboration and allowed for investigator’s judgement for collection of information about the three zoonoses of interest which are characterised by multiple allied risk factors for transmission. Our inclusion of brucellosis and Q fever as the diseases of interest approach has obvious marginal cost advantages over study designs which are confined to one disease of interest.

Householder and animal *Brucella* seroprevalences were 5.2% (95% CI 1.8, 14.3) and 1.3% (95%CI 0.7, 2.2) respectively with 15.7% of households having seropositive householders and 12.3% seropositive animals. There was evidence of clustering of infection in animals within a household and Kuchi village animals were more likely to be seropositive than those in sedentary villages (Table 1).

The evidence of clustering is presented in the moderate level of household variance (Table 1)

The relatively constant prevalence over all age bands in householders (Fig 1B) indicates early age exposure to infection and contrasts with a finding of no seropositive animals in the 12–23 month age group of animals (Fig 3B). Humans are susceptible to infection at all ages, whereas with the exception of latent infection, successful infection in animals mainly occurs during pregnancy and most of the animals in the 12–23 month age band would have been either non-pregnant or in early first-parity pregnancy at the time of sampling. Seroprevalences of 1.4% in sheep and 1.5% in goats are highly indicative of *B*. *melitensis* infection for which the predominant reservoir hosts are small ruminants. Serology cannot distinguish Brucella species and the single test positive in cattle could be due to either *B*. *melitensis* or *B*. *abortus*.

The high householder and animal *C*. *burnetii* seroprevalences of 63.9% (95%CI 57, 70.3) and 43.4% (95%CI 34.7, 52.5) respectively with almost all households (97.5% and 98.5%) having evidence of exposure to infection in householders and household livestock were unexpected findings. Prevalences varied among villages but not between Kuchi and sedentary villages or between sheep and goats. As with *Brucella* the prevalence was markedly lower in cattle than in sheep and goats and there was evidence of early age exposure to infection in householders and animals.

Participation was voluntary and householders were not randomly selected but given the sample size of five and the average Afghan household size of 7.6 and our restriction of ages to between 9 and 60 years of age it was interesting that more female than male householders participated. Gender inequality is an issue for health care in Afghanistan and we suspect that one of the reasons for relatively more females participating was that they welcomed the opportunity afforded by the visits of health workers to their homes. Also females are at home most of the time and some males may have been absent due to work commitments. Given the sample size of five persons per household we consider any bias from possible over-representation of persons who were unwell to be unlikely and of no consequence. The overall seroprevalence estimates for householders and animals provide the best available estimates of risk of exposure to infection in the populations of interest with the caution that the estimates are conditional on uncertainties regarding the operating characteristics of the commercial ELISAs which were used and the prevalence of false positives from other sources of non-specific reactions. The precision of the estimate for householders is less certain than the estimate for animals because it is not known for how long *C*. *burnetii* and *Brucella* titres persist in humans. Sheep and goats remain infected after initial infection with *B*. *melitensis* and are serology test positive for life [12] unlike humans where infection, apart from a small proportion of chronic relapsing cases, is usually self-limiting. The nature of *C*. *burnetii* infection is probably similar; infections in animals may persist for long periods [13,14] and most human infections are self-limiting.

We considered household prevalences derived by the study to be the most informative measures of risk of exposure to infection. However, the prevalences of households with evidence of exposure to *Brucella* infection in animals and householders are biased downwards to an unknown extent because only ten animals were tested in each household and the prevalence of *Brucella* test positive animals was low. The bias was of no consequence for *C*. *burnetii* because of the high proportions of households with test positive householders and animals.

Confidence intervals of prevalence estimates were quite wide because multistage sampling with village as the primary sampling unit was done and this was accounted for in the analysis. True random sampling at each level (village, household and individual) with participation of 94% of selected households ensured that these are valid estimates. We also remind readers here that the study population consisted of households with animals so the estimates are only valid for that sub-population and not for the general population.

Many of the factors examined were village- or household-level factors for which the effective sample sizes were the numbers of villages and households. Despite this limitation and the study’s reliance on serology with no information as to when infection occurred, the multivariable analyses were performed in an attempt to identify at least some of the risk factors for infection in householders. The fact that the only factor identified for *Brucella* seropositivity in householders was *Coxiella* seropositivity may have been due to its low prevalence which would have limited the power of the analysis for this infection. However, male gender, milking sheep, drinking raw milk, treating animals for ticks and *Brucella* seropositivity were identified for *C*. *burnetii* (see Table 4) and these are in accord with current understanding of the disease which considers the main routes of infection to be via infected milk and birth products and inhalation of contaminated dust. The nature of *C*. *burnetii* infections has been well described in recent comprehensive reviews [15,16,17]. The organism can survive well for extremely long times in its desiccated spore-like form in dust; cattle sheep and goats are the main reservoirs for infections and ticks may be the natural primary reservoir host[17]. *C*. *burnetii* infection is asymptomatic in 50% to 60% of acute infections and its clinical polymorphism makes diagnosis difficult[17]. Abortions may occur in pregnant women and the chronic form which develops in about 5% of infections manifests as endocarditis, hepatitis and pneumonia. Diagnosis in rural communities in Afghanistan is particularly challenging because of poor access to competent and well-equipped health services and laboratories and competition from traditional healers in rural villages. A Disease Early Warning System (DEWS) for surveillance, investigation and reporting of infectious diseases in Afghanistan is operated by the surveillance department of the Afghanistan National Public Health Institute within the Ministry of Public Health. Q fever is not on the DEWS list of priority diseases and clinical syndromes but this study has greatly increased awareness of its importance among public health and veterinary authorities. Although *C*. *burnetii* infections in animals may result in abortion and shedding in birth products and milk, most infections are asymptomatic and differentiation from other causes of abortion which are known or suspected to be endemic in ruminants in Afghanistan is virtually impossible for reasons similar to those which prevail in the public health sector.

*Brucella* seropositivity (higher risk) and Kuchi flocks (lower risk) were apparently associated with abortion in animals (Table 3) but no associations were found for *C*. *burnetii* seropositivity and abortions. Both infections may cause abortions in animals [16,18] but it is likely that a high proportion of animals of breeding age would be immune to *C*. *burnetii* because of a high level of exposure before pregnancy unlike conditions for infection which would apply for *Brucella*. We consider the associations and the difference between *Brucella* and *C*. *burnetii* seropositivity to be interesting and worth reporting with the caution that the links with abortion should only be regarded as associations and not as evidence of causality because the timing of infection with regard to abortion was not possible to determine in a cross-sectional study.

The KAP component of the study produced information about the prevalence of risky practices for infection within households and the analysis of the evidence of exposure to *Brucella* in householders or in their livestock identified high risk households as Kuchi with ≤4 rooms in their house and not owning land (Table 5). Activities which have been shown to be risky for zoonotic transmission of *Brucella* in other resource-limited countries include rural residence, contact with infected animals at parturition, consumption of unpasteurised milk and dairy products, high risk occupations such as veterinarians and slaughterhouse workers [19,20,21,22,23] and our study recorded disturbingly high prevalences of all of these activities among householders.

The study produced useful information which can be incorporated into the design of control strategies and was a catalyst for a nation-wide serological survey of brucellosis in animals and the introduction of Rev1 vaccination of small ruminants by private veterinary units in high prevalence districts. Rev1 vaccination is the most important component of a *B*. *melitensis* control program but its success depends on adequate coverage, identification of vaccinates, timely and adequate supply of quality assured vaccine, awareness in rural communities, movement controls and surveillance; all conditions which need to be maintained for many years for sustainability. These conditions are extremely difficult to achieve in the uneasy and unsafe environment which prevails in Afghanistan with almost total reliance on often poorly coordinated donor support from many NGOs and international organisations such as the FAO and the World Health Organisation. Control of *C*. *burnetii* infections in humans and animals is difficult even in well-resourced countries [24,25] but the outlook for its control in Afghanistan is particularly bleak. The success which *C*. *burnetii* enjoys in the Afghanistan environment is testament to the husbandry and livestock management practices which favour its long term survival, exposure at a young age and highly efficient modes of transmission. Awareness campaigns aimed at educating householders and livestock owners about avoidance of risky practices offer some hope and benefits for brucellosis and echinococcosis but are likely to have little impact on *C*. *burnetii*. A high risk for Echinococcosis was identified by a high prevalence (62%) of feeding guts from slaughtered sheep or goats to dogs.

In summary the study alerted authorities to a hitherto unrecognised high prevalence of *C*. *burnetii* infections, acted as a catalyst for Rev1 vaccination of sheep and goats, demonstrated the benefits of a coordinated approach and fostered a better understanding of the nature of infection in different hosts and of the constraints for control faced by government services. A notable feature of the study was the enthusiasm and interest displayed by all of the participants throughout, from heads of government services to field personnel and villagers. The high level of interest displayed by the villagers was illustrated by the very high participation rate (93.6%) of the randomly selected households which were invited to participate. Livestock owners regard zoonoses as adversities that affect their livestock and members of their households and do not partition them separately as medical or veterinary problems. Control programs need to take that perception into account and wherever possible avoid vesting ownership separately into veterinary or public health agencies. Education programs targeted to the large numbers of Community Health Centres (385), Basic Health Centres (813) and Veterinary Field Units (1,200–1,500) in the Republic have the potential to create awareness about risky practices for zoonoses among livestock owners and householders at village level and thereby reduce the incidence of disease.

## Supporting Information

S1 Checklist STROBE Checklist. (DOC)

S1 File Animal test recording form. (DOCX)

S2 File Blood sample information form. (DOCX)

S3 File Householder KAP survey. (DOC)

S4 File National Multisectoral Assessment on Kuchi. (DOC)
