# Supplementary material for: Brucellosis and Coxiella burnetii Infection in Householders and Their Animals in Secure Villages in Herat Province, Afghanistan: A Cross-Sectional Study
Source: PLoS Negl Trop Dis. 2015 Oct 20;9(10):e0004112. doi: 10.1371/journal.pntd.0004112 (PMC4618140; doi:10.1371/journal.pntd.0004112)
Supplement: S1 File — (DOCX) [file pntd.0004112.s002.docx]

# ANIMAL TEST RECORDING FORM

| **Name of VFU taking the sample** |  | **Date of sampling** |  | **Village code** |  |
| --- | --- | --- | --- | --- | --- |

| **HH ID** | **Owner name** | **Animal Specification** | **Age (months)** | **Species**  **1=sheep**  **2=goat**  **3=cattle** | **Animal ever aborted?**  **0= No**  **1= Yes**  **2=don’t know** | **Brucella**  **RBT**  **0= –ve**  **1= +ve** | **Brucella**  **ELISA**  **0 = –ve**  **1 = +ve** | **CCHF**  **titre** | **Q fever**  **titre** |
| --- | --- | --- | --- | --- | --- | --- | --- | --- | --- |
|  |  |  |  |  |  |  |  |  |  |
|  |  |  |  |  |  |  |  |  |  |
|  |  |  |  |  |  |  |  |  |  |
|  |  |  |  |  |  |  |  |  |  |
|  |  |  |  |  |  |  |  |  |  |
|  |  |  |  |  |  |  |  |  |  |
|  |  |  |  |  |  |  |  |  |  |
|  |  |  |  |  |  |  |  |  |  |
|  |  |  |  |  |  |  |  |  |  |
|  |  |  |  |  |  |  |  |  |  |
|  |  |  |  |  |  |  |  |  |  |
|  |  |  |  |  |  |  |  |  |  |
|  |  |  |  |  |  |  |  |  |  |
|  |  |  |  |  |  |  |  |  |  |
|  |  |  |  |  |  |  |  |  |  |
|  |  |  |  |  |  |  |  |  |  |
|  |  |  |  |  |  |  |  |  |  |
|  |  |  |  |  |  |  |  |  |  |
|  |  |  |  |  |  |  |  |  |  |
|  |  |  |  |  |  |  |  |  |  |

| Date + time received at PVL | |  | Date + time sent to CVDRL |  | Date + time received at CVDRL |  |
| --- | --- | --- | --- | --- | --- | --- |
|  |  |  |  |  |  | **Date of testing**  ***THIS SECTION TO BE COMPLETED BY THE LABS*** |
|  |  |  |  | Brucella – RBT | |  |
|  |  |  |  | Brucella – ELISA | |  |
|  |  |  |  | CCHF | |  |
|  |  |  |  | Q fever | |  |
