# Supplementary material for: Brucellosis and Coxiella burnetii Infection in Householders and Their Animals in Secure Villages in Herat Province, Afghanistan: A Cross-Sectional Study
Source: PLoS Negl Trop Dis. 2015 Oct 20;9(10):e0004112. doi: 10.1371/journal.pntd.0004112 (PMC4618140; doi:10.1371/journal.pntd.0004112)
Supplement: S2 File — (DOCX) [file pntd.0004112.s003.docx]

This form should be filled out for every member of the household 8 years or older to 60 years.

No blood will be taken from children <8 years of age.

| Household ID |  | Individual ID |  | Q1. Name |  |
| --- | --- | --- | --- | --- | --- |

| Q2. Age |  | years | Q3. Gender | Male |  | Female |  |  | Q4. Are you married? | Yes |  | No |  |
| --- | --- | --- | --- | --- | --- | --- | --- | --- | --- | --- | --- | --- | --- |

**If not married go to Q8. If married go to Q5.**

| Q5 How many children do you have? | If participant is male do not ask Q6 & 7, go to Q8 | Q6. Are you pregnant? | Yes |  | No |  |
| --- | --- | --- | --- | --- | --- | --- |

| Q7. Have you ever experienced an abortion? | Yes |  | No |  |  | Q8. Are you sick now? | Yes |  | No |  |
| --- | --- | --- | --- | --- | --- | --- | --- | --- | --- | --- |

**If No to Q8 go to Q10.**

| Q9. Do you have any of the following symptoms?  ***Interviewer to specify the options*** | Body pain |  | 1 | Fever |  | 6 |
| --- | --- | --- | --- | --- | --- | --- |
|  | Weakness |  | 2 | Joint pain |  | 7 |
|  | Loss of appetite |  | 3 | Joint swelling |  | 8 |
|  | Sweating |  | 4 | Headaches |  | 9 |
|  | Weight loss |  | 5 |  |  |  |

| Q10. Which in this list best describes your **main** occupation  ***Tick only one option***  ***If not on the list please specify at Other occupation*** | Farmer or farm worker | |  | 1 |
| --- | --- | --- | --- | --- |
|  | Housewife | |  | 2 |
|  | Student | |  | 3 |
|  | Person not working | |  | 4 |
|  | Government or Local district administration worker | |  | 5 |
|  | Clerical worker | |  | 6 |
|  | Teacher | |  | 7 |
|  | Trader of meat products | |  | 8 |
|  | Trader of dairy products | |  | 9 |
|  | Veterinarian | |  | 10 |
|  | Animal health worker (vet assistant, paravet, BVW, LEW) | |  | 11 |
|  | Abattoir worker | |  | 12 |
|  | Milk plant worker | |  | 13 |
|  | Shepherd | |  | 14 |
|  | Human health worker - nurse | |  | 15 |
|  | Human health worker - doctor | |  | 16 |
|  | Human health worker - CHW | |  | 17 |
|  | Other occupation - specify |  | | |

| Q11. Which of the tasks in this list do you do?  ***Tick all options that are mentioned*** | Milk cows |  | 1 |
| --- | --- | --- | --- |
|  | Milk sheep |  | 2 |
|  | Milk goats |  | 3 |
|  | Prepare milk products – cheese |  | 4 |
|  | Prepare milk products – butter |  | 5 |
|  | Prepare milk products – yoghurt |  | 6 |
|  | Prepare milk products – other |  | 7 |
|  | Slaughter animals and prepare meat |  | 8 |
|  | Feed young animals |  | 9 |
|  | Clean out sheep, goat or cattle pens |  | 10 |
|  | Help at birthing time |  | 11 |
|  | Look after animals at communal grazing |  | 12 |
|  | Shearing sheep or goats |  | 13 |
|  | Remove ticks from animals |  | 14 |
|  | Treat animals for ticks with insecticide |  | 15 |

| Q12. Do you sometimes drink milk that has not been boiled? | Yes |  | No |  |
| --- | --- | --- | --- | --- |

| Blood volume collected ≥ 7ml? |  | Date + time received PVL |  | Date + time sent from PVL to Kabul |  | Date + time received CPHL | ***THIS SECTION TO BE COMPLETED BY THE LABS*** |
| --- | --- | --- | --- | --- | --- | --- | --- |
| Good serum separation? |  |  |  |  |  |  |  |

| Brucella RBT  (0 = -ve, 1 = +ve) |  | Brucella ELISA  (0 = -ve, 1 = +ve) |  | CCHF titre |  | Q fever |  |
| --- | --- | --- | --- | --- | --- | --- | --- |
