# Supplementary material for: Brucellosis and Coxiella burnetii Infection in Householders and Their Animals in Secure Villages in Herat Province, Afghanistan: A Cross-Sectional Study
Source: PLoS Negl Trop Dis. 2015 Oct 20;9(10):e0004112. doi: 10.1371/journal.pntd.0004112 (PMC4618140; doi:10.1371/journal.pntd.0004112)
Supplement: S3 File — (DOC) [file pntd.0004112.s004.doc]

# HOUSEHOLDER BRUCELLOSIS KNOWLEDGE ATTITUDES PRACTICES SURVEY

| **INFORMED CONSENT** | **Village and household code** |  |
| --- | --- | --- |

| Hello. My name is ___________________ and I am working with the Department of Public Health. We are conducting a survey and would appreciate your participation. I would like to ask you about your family’s health and the health services available in your area. This information will help us to plan and improve health programs in your village, and in other parts of Afghanistan. The survey usually takes about 20 minutes to complete. Whatever information you provide will be kept strictly confidential and will not be shown to other persons.  Participation in this survey is voluntary and you can choose not to answer any individual question or all of the questions. However, we hope that you will participate in this survey since your views are important.  At this time, do you want to ask me anything about the survey?  ***Signature of Interviewer****:* _________________________________***Date****:* ____ / ____ / ____ |
| --- |

### GENERAL INFORMATION

| Q1. Gender | Male |  | Female |  |  | Q2. How old are you? |  | years |
| --- | --- | --- | --- | --- | --- | --- | --- | --- |

| Q3. What is your marital status  ***Tick only one option*** | Married |  | 1 |
| --- | --- | --- | --- |
|  | Married *(spouse away)* |  | 2 |
|  | Widowed |  | 3 |
|  | Divorced |  | 4 |
|  | Single *(never married)* |  | 5 |

| Q4. How many persons are in your household? |  | persons |
| --- | --- | --- |

| Q5. How many rooms are there in your house? |  | rooms |
| --- | --- | --- |

| Q6. What is the makeup of your household  ***Tick only one option*** | Couple without children |  | 1 |
| --- | --- | --- | --- |
|  | Couple with one or more children |  | 2 |
|  | Couple with one or more children and parents/parents in law |  | 3 |
|  | Couple with one or more children and parents/parents in law and brothers/sisters or brothers/sisters in law |  | 4 |
|  | Couple with married children |  |  |

| Q7. Does your family own land? | Yes |  | No |  |
| --- | --- | --- | --- | --- |

| Q8. How many female cattle of breeding age do you have? |  | female cattle 2 years of age or older |
| --- | --- | --- |

IF answer to Q 8 is zero go to Q 10

| Q9. Do you milk the cattle? | Yes |  | No |  |
| --- | --- | --- | --- | --- |

| Q10. How many female sheep of breeding age do you have? |  | female sheep 1 year of age or older |
| --- | --- | --- |

IF answer to Q 10 is zero go to Q 12

| Q11. Do you milk the sheep? | Yes |  | No |  |
| --- | --- | --- | --- | --- |

| Q12. How many female goats of breeding age do you have? |  | female goats 1 year of age or older |
| --- | --- | --- |

IF answer to Q 12 is zero go to Q 14

| Q13. Do you milk the goats? | Yes |  | No |  |
| --- | --- | --- | --- | --- |

| Q14. Are any of the following available in your household?  ***Tick all options that are mentioned*** | Electricity |  | 1 |
| --- | --- | --- | --- |
|  | Radio |  | 2 |
|  | TV |  | 3 |
|  | Refrigerator |  | 4 |
|  | Mobile phone |  | 5 |
|  | None of the above |  | 6 |

| Q15. Does your family own any of the following?  ***Tick all options that are mentioned*** | Bicycle |  | 1 |
| --- | --- | --- | --- |
|  | Horse |  | 2 |
|  | Donkey |  | 3 |
|  | Motorcycle |  | 4 |
|  | Car |  | 5 |
|  | Truck |  | 6 |
|  | Tractor |  | 7 |
|  | None of the above |  | 8 |

| Q16. Which in this list best describes your **main** occupation  ***Tick only one option***  ***If not on the list please specify at Other occupation*** | Farmer or farm worker | |  | 1 |
| --- | --- | --- | --- | --- |
|  | Housewife | |  | 2 |
|  | Student | |  | 3 |
|  | Person not working | |  | 4 |
|  | Government or Local district administration worker | |  | 5 |
|  | Clerical worker | |  | 6 |
|  | Teacher | |  | 7 |
|  | Trader of meat products | |  | 8 |
|  | Trader of dairy products | |  | 9 |
|  | Veterinarian | |  | 10 |
|  | Animal health worker (vet assistant, paravet, BVW, LEW) | |  | 11 |
|  | Abattoir worker | |  | 12 |
|  | Milk plant worker | |  | 13 |
|  | Shepherd | |  | 14 |
|  | Human health worker - nurses | |  | 15 |
|  | Human health worker - doctor | |  | 16 |
|  | Human health worker - CHW | |  | 17 |
|  | Other occupation – specify |  | | |

| Q17. What is your educational status?  ***Tick only one option*** | Primary school – Class 1 to Class 6 |  | 1 |
| --- | --- | --- | --- |
|  | Secondary school - Class 7 to Class 9 |  | 2 |
|  | Secondary school - Class 10 to Class 12 |  | 3 |
|  | Higher education |  | 4 |
|  | None |  | 5 |

| Q18. In the past three months have you heard or seen any health education messages? | Yes |  | No |  | Not sure |  |
| --- | --- | --- | --- | --- | --- | --- |

**If you answer NO or not sure to Q18, go to Q20**

| Q19. Where did you hear or see these health messages?  ***Tick all options that are mentioned*** | Human health worker | |  | 1 |
| --- | --- | --- | --- | --- |
|  | Veterinary field unit (VFU) | |  | 2 |
|  | Animal health extension worker | |  | 3 |
|  | School | |  | 4 |
|  | International organization | |  | 5 |
|  | Radio | |  | 6 |
|  | TV | |  | 7 |
|  | Posters | |  | 8 |
|  | Newspaper | |  | 9 |
|  | Relative or friend | |  | 10 |
|  | Mosque | |  | 11 |
|  | Tea house | |  | 12 |
|  | Not sure | |  | 13 |
|  | Other – specify |  | | |

### BRUCELLOSIS

| Q20. Which of the following diseases can be transmitted from animals to humans  ***Tick all options that are mentioned*** | Brucellosis |  | 1 |
| --- | --- | --- | --- |
|  | Typhoid |  | 2 |
|  | Anthrax |  | 3 |
|  | Malaria |  | 4 |
|  | Mange |  | 5 |
|  | Hepatitis |  | 6 |
|  | TB |  | 7 |
|  | Rabies |  | 8 |
|  | Avian influenza |  | 9 |
|  | Foot and mouth disease |  | 10 |
|  | Entero-toximia |  | 11 |
|  | Don’t know any |  | 12 |
|  | Other (specify) |  |  |

**If doesn’t know brucellosis go to Q36**

| Q21. How do you think a person gets infected with brucellosis?  ***Do not read out or prompt for an answer.***  ***Tick all options that are mentioned*** | Coughing | |  | 1 |
| --- | --- | --- | --- | --- |
|  | Sneezing | |  | 2 |
|  | Direct contact with other persons | |  | 3 |
|  | Sharing dishes with other persons | |  | 4 |
|  | Excretions of animals | |  | 5 |
|  | Direct contact with animals | |  | 6 |
|  | Dairy products | |  | 7 |
|  | Meat and meat products | |  | 8 |
|  | Meat products | |  | 9 |
|  | Caring for animals | |  | 10 |
|  | Other foodstuffs (eggs) | |  | 11 |
|  | Other foodstuffs (fish) | |  | 12 |
|  | Don’t know | |  | 13 |
|  | Other – specify |  | | |

| Q22. What are the symptoms of brucellosis?  ***Do not prompt for this question***  ***Tick all options that are mentioned*** | Body pain | |  | 1 |
| --- | --- | --- | --- | --- |
|  | Weakness | |  | 2 |
|  | Loss of appetite | |  | 3 |
|  | Sweating | |  | 4 |
|  | Weight loss | |  | 5 |
|  | Fever | |  | 6 |
|  | Joint pains | |  | 7 |
|  | Joint swelling | |  | 8 |
|  | Headaches | |  | 9 |
|  | Don’t know | |  | 10 |
|  | Other – specify |  | | |

| Q23. Do you think brucellosis is an infectious disease? | Yes |  | No |  | Don’t know |  |
| --- | --- | --- | --- | --- | --- | --- |

| Q24. Do you think a person with brucellosis can be cured? | Yes |  | No |  | Don’t know |  |
| --- | --- | --- | --- | --- | --- | --- |

IF answer to Q 24 is no or don’t know go to Q 26

| Q25. How long does it take for a person with brucellosis to be cured?  ***Do not prompt for this question***  ***Tick only one option*** | <2 weeks |  | 1 |
| --- | --- | --- | --- |
|  | 2-4 weeks |  | 2 |
|  | 1-2 months |  | 3 |
|  | 3-6 months |  | 4 |
|  | >6 months-1year |  | 5 |
|  | >1year |  | 6 |
|  | Don’t know |  | 7 |

| Q26. What can you do to prevent brucellosis?  ***Do not read out or prompt for an answer.***  ***Tick all options that are mentioned*** | Cook meat | |  | 1 |
| --- | --- | --- | --- | --- |
|  | Boil milk before drinking | |  | 2 |
|  | Boil milk used for making dairy products | |  | 3 |
|  | Keep infected animals separate from healthy animals | |  | 4 |
|  | Vaccinate cattle, sheep, goats | |  | 5 |
|  | Clean and Disinfect barns | |  | 6 |
|  | Bury or destroy placenta after abortion or giving birth | |  | 7 |
|  | Wear gloves during assistance of animal at birthing time | |  | 8 |
|  | Wash/disinfect hands after assistance to animals | |  | 9 |
|  | Don’t know | |  | 10 |
|  | Other - specify |  | | |

| Q27. To whom or where would you go for advice or treatment for brucellosis  ***Do not read out or prompt for an answer.***  ***Tick all options that are mentioned*** | health facility | |  | 1 |
| --- | --- | --- | --- | --- |
|  | Private doctor | |  | 2 |
|  | Traditional healer | |  | 3 |
|  | CHW | |  | 4 |
|  | Other source of advice or treatment – specify |  | | |

| Q28. During the past year did any person in your household have brucellosis? | Yes |  | **🡺 Q 29** |
| --- | --- | --- | --- |
|  | No |  | **🡺 Q 32** |
|  | Don’t know |  | **🡺 Q 32** |

| Q29. Who in your household had brucellosis in the past year?  ***Write down the number of persons which were ill next to the particular age categories*** | Child < 5 years |  | 1 |
| --- | --- | --- | --- |
|  | Child 5-14 years |  | 2 |
|  | Adolescent 15-19 years |  | 3 |
|  | Adult 20-49 years |  | 4 |
|  | Adult ≥ 50 years |  | 5 |

| Q30. If any person in your household had brucellosis in the past 12 months, did they take care of animals during birthing? | Yes |  | No |  | Don’t know |  |
| --- | --- | --- | --- | --- | --- | --- |

| Q31. Were they treated for brucellosis at home, in the health care centre, elsewhere or not treated at all?  ***Tick all options that are mentioned*** | Home | |  | 1 |
| --- | --- | --- | --- | --- |
|  | Health care centre | |  | 2 |
|  | Not treated | |  | 3 |
|  | Don’t know | |  | 4 |
|  | Elsewhere - specify |  | | |

|  |  | | | |  | |  | |
| --- | --- | --- | --- | --- | --- | --- | --- | --- |
|  |  |  |  |  |  |  |  |  |
|  |  |  |  |  |  |  |  |  |
|  |  |  |  |  |  |  |  |  |
|  |  |  |  |  |  |  |  |  |
| Q32. If your female sheep, goats or cattle have brucellosis what signs of the disease would you see?  Do not read out  ***Tick all options that are mentioned*** | | Blood in milk | | | |  | | 1 |
|  |  | Abortion (death of fetus) | | | |  | | 2 |
|  |  | Decreased milk production | | | |  | | 3 |
|  |  | Sterility | | | |  | | 4 |
|  |  | Retained placenta (afterbirth) | | | |  | | 5 |
|  |  | Lameness | | | |  | | 6 |
|  |  | Mastitis (udder inflammation) | | | |  | | 7 |
|  |  | Small & weak newborn | | | |  | | 8 |
|  |  | Stop milking | | | |  | | 9 |
|  |  | No signs at all | | | |  | | 10 |
|  |  | Don’t know | | | |  | | 11 |
|  |  | Other – specify | |  | | | | |

| Q33. If a male donkey or horse has brucellosis what signs of the disease would you see?  ***Tick all options that are mentioned*** | High fever |  | 1 |
| --- | --- | --- | --- |
|  | Lameness |  | 2 |
|  | Hard swelling around joints |  | 3 |
|  | Large lumps on the neck |  | 4 |
|  | Coughing |  | 5 |
|  | Not eating |  | 6 |
|  | Don’t know |  | 7 |

| Q34. Do any of your animals have brucellosis? | Yes |  | No |  | Dont know |
| --- | --- | --- | --- | --- | --- |

If no or don’t know go to Q 36

| Q35. If yes do you handle those animals or their animal products in a special way? | Yes |  | No |  | Don’t know |  |
| --- | --- | --- | --- | --- | --- | --- |

| Q36. How do you use the milk that you get from your cattle sheep or goats?  ***Do not prompt for this question***  ***Tick all answers that are mentioned*** | After boiling | |  | 1 |
| --- | --- | --- | --- | --- |
|  | Use without boiling | |  | 2 |
|  | Do not use milk products | |  | 3 |
|  | Sell or trade or gift | |  | 4 |
|  | Other – specify |  | | |

| Q37. What milk products do you make at home from milk that has not been boiled?  ***Tick all options that are mentioned*** | Cheese | |  | 1 |
| --- | --- | --- | --- | --- |
|  | Cream | |  | 2 |
|  | Butter | |  | 3 |
|  | Yoghurt | |  | 4 |
|  | Do not use milk that has not been boiled | |  | 5 |
|  | Other – specify |  | | |

| Q38. Who in your household does the milking?  ***Insert relationship of family member, e.g. mother, sister, daughter, brother*** |  |
| --- | --- |
|  |
|  |
|  |

| Q39. Do those persons who do the milking take any special precautions after milking?  ***Do not prompt for this question*** | Wash hands with soap and water | |  | 1 |
| --- | --- | --- | --- | --- |
|  | No special precautions | |  | 2 |
|  | Other – specify |  | | |

| Q40. Who in your household cares for the animals at birthing?  ***Insert relationship of family member, e.g. mother, sister, daughter, brother*** |  |
| --- | --- |
|  |
|  |
|  |

| Q41. Do those persons take any special precautions when they handle the animals at birthing?  ***Do not prompt for this question***  ***Tick all options that are mentioned*** | Wear gloves | |  | 1 |
| --- | --- | --- | --- | --- |
|  | Wash hands with soap and water | |  | 2 |
|  | Clean and disinfect the birth pens | |  | 3 |
|  | Nothing | |  | 4 |
|  | Other – specify |  | | |

| Q42. What do you do with the afterbirth or aborted fetus?  ***Do not prompt for this question***  ***Tick all options that are mentioned*** | Bury it | |  | 1 |
| --- | --- | --- | --- | --- |
|  | Burn it | |  | 2 |
|  | Feed it to the dogs | |  | 3 |
|  | Throw it in the river | |  | 4 |
|  | Throw in the open environment | |  | 5 |
|  | Other – specify |  | | |

| Q43. What do you do with the guts after you have slaughtered a sheep or goat?  ***Do not prompt for this question***  ***Tick all options that are mentioned*** | Bury it | |  | 1 |
| --- | --- | --- | --- | --- |
|  | Burn it | |  | 2 |
|  | Feed it to the dogs | |  | 3 |
|  | Throw it in the river | |  | 4 |
|  | Throw in the open environment | |  | 5 |
|  | Other – specify |  | | |

| Q44. Have any of your animals been vaccinated against any diseases in the past 12 months? | Yes |  | No |  | Don’t know |  |
| --- | --- | --- | --- | --- | --- | --- |
| If answer to Q 44 is don’t know go to Q 46 | | | | | | |
| Q 45, If yes what are they?  Tick all that apply | Brucellosis | | | |  | 1 |
|  | Anthrax | | | |  | 2 |
|  | Enterotoxin | | | |  | 3 |
|  | Foot and Mouth Disease | | | |  | 4 |
|  | Sheep Goat Pox | | | |  | 5 |
|  | CCPP | | | |  | 6 |
|  | Don’t know | | | |  | 7 |
|  | Other specify | | | |  |  |

| Q46. From where do you most often get your milk and dairy products?  ***Tick only one option*** | Own animals | |  | 1 |
| --- | --- | --- | --- | --- |
|  | Local market | |  | 2 |
|  | Local store | |  | 3 |
|  | Neighbours | |  | 4 |
|  | Other – specify |  | |  |

| Q47. Please list other illnesses that members of your household suffered from during the past 12 months |  |
| --- | --- |
|  |
|  |
|  |
|  |

***“This is the end of the interview. Thank you very much for assisting us with this important survey. Remember that your answers will be kept confidential. If you have questions about any of the topics we discussed, I can try to answer them now or refer you to someone else.***

***Here is some information about brucellosis that will help to protect you and members of your household from this disease.” Leave educational pamphlets about brucellosis.***
