# Supplementary material for: Brucellosis and Coxiella burnetii Infection in Householders and Their Animals in Secure Villages in Herat Province, Afghanistan: A Cross-Sectional Study
Source: PLoS Negl Trop Dis. 2015 Oct 20;9(10):e0004112. doi: 10.1371/journal.pntd.0004112 (PMC4618140; doi:10.1371/journal.pntd.0004112)
Supplement: S4 File — (DOC) [file pntd.0004112.s005.doc]

**National Multi sectoral Assessment on Kuchi**

**Main findings**

**Author: Frauke de Weijer**

With support from Andrew Pinney, Amanullah Assil, Zuhoor Mehri and Shamim Kabuli

**Date:** May 2005

**Table of Content**

1. Introduction

2. Methodology

3. Kuchi categories

4. Demographic patterns

5. Pasture

6. Land ownership

7. Livestock ownership

8. Water

9. Labour and other sources of income

10. Health care

11. Education

12. Mines

13. Intervention priorities

14. Conclusions and recommendations

Annex I: Translation of months

Annex II: Regional categories of provinces

Annex III: Questionnaire

Annex IV: Province overviews

Annex V: Tables

**1. Introduction**

Kuchi is a term that is generally used to describe the transhumant or nomadic pastoralists of Afghanistan. In fact it is a term that can cause confusion, since it refers to both a lifestyle (migratory), a production mode (livestock dependent), and a cultural identity. The more appropriate term to use for this group of people is ‘pastoralists’, which refers to the livestock production mode. Pastoralism is a social and economic system based on the raising and herding of livestock.

However in reality, many ‘Kuchi’ may have settled decades ago, own land or large transportation companies, and still refer to themselves as ‘Kuchi’. Also those that have lost their livestock during the years of war or the recent drought and have been forced to settle (temporarily?) still refer to themselves as Kuchi, and have as yet not been able to establish an alternative livelihood.

Primary data on Kuchi is scarce. Experience has demonstrated the constraints encountered when trying to include this group in assessments geared towards settled communities. Census exercises carried out in the past have always struggled with the nomads, and population figures have been estimates at best. Data on Kuchi have been collected during the 2003 NRVA exercise, but in relatively low numbers (83 communities) making aggregation and extrapolation difficult. In this report, data from NMAK and from NRVA will be compared, if possible.

This lack of information has constrained the development of strategies aimed at supporting the Kuchi and as a result the Kuchi have not received equitable access to services and reconstruction efforts. This was recognized by the cabinet in late 2003 and an inter-ministerial commission on Kuchi was established to mitigate this. The inter-ministerial commission on Kuchi was supported by a technical Kuchi Vulnerability Committee, a coordination body established under the Ministry of Frontiers and Tribal Affairs, with stakeholders from all relevant ministries or other organizations. Members of this Committee from the sphere of health, agriculture, education, and others all expressed the urgent need to obtain more information on the Kuchi, to enable them to develop programs and allocate resources in a fair and appropriate manner. This demand led to the important decision that a multi-sectoral assessment shall be implemented as soon as possible; the National Multi-sectoral Assessment on Kuchi (NMAK).

This assessment was conducted in early 2004, just before the onset of spring migration. The lead was taken by the Ministry of Rural Rehabilitation and Development, in close collaboration with Ministry of Frontiers and Tribal Affairs and the Central Statistics Office.

***Objective***

The objective of the National Multi-sectoral Assessment on Kuchi was to obtain baseline demographic information on pastoralist communities, and their current access to service related infrastructure, in order to provide the government with the necessary data to support programme design.

It is not the objective of this assessment to be a full census of the Kuchi population of Afghanistan, even though it does aim at providing estimates. It is also not meant to describe and compare vulnerability levels, for which the NRVA a better instrument.

The NMAK data is essentially primary data on Kuchi; on their demographic patterns and their current access to services. Areas of concern can be indicated on the basis of this data, and basic recommendations on issues that require attention. However, full policy recommendations required more than primary data alone, but it is hoped that this document can serve as the basis.

**2. Methodology**

Representatives of the Inter-ministerial Commission on Kuchi, and other interested members of the Kuchi Vulnerability Committee were requested to formulate their information needs on Kuchi. Since the specific objective of this assessment was to fill information needs for programming and planning, and not directly for a vulnerability assessment, the members were requested to formulate their questions accordingly.

These information needs were compiled and analyzed by the Assessment Management Team; a team formed from members of the MRRD Pastoralist Support Unit (Ms. Frauke de Weijer, Mr. Amanullah Assil and Mr. Zuhoor Mehri), the MRRD Vulnerability Analysis Unit (Mr. Andrew Pinney and Mr. Tariq Wardak), and staff from the WFP Vulnerability Analysis and Mapping (VAM) unit (Mr. Saadat Khan) seconded by WFP to the assessment. Furthermore, direct cooperation was sought with CSO (Mr. Sami Nabi), to benefit from their expertise in assessments.

Since the most pressing information need was an understanding of the demographic patterns of the Kuchi, *‘how many are where when?’*, an assessment based upon a sampling framework was not recommended. Instead an assessment was required which covered all Kuchi in the country. For reasons of manageability, the chosen unit of observation was the ‘community’.

***Definitions***

As explained above, the term ‘***Kuchi***’ is likely to cause confusion and a blurring of lines between different types of people. Many ‘Kuchi’ that settled a long time, if not decades ago, still consider themselves Kuchi. It is as much a lifestyle as it is a cultural identity. To differentiate between these people, and to ensure that nation-wide the same definition is used, the following definition was used in the NMAK:

***A Kuchi is either migratory, or has settled recently due to loss of livestock during the last drought.***

A ***‘community’***, the basis unit of observation, was defined as ‘a group of households’ that stay in one area, and have the same winter- and summer grazing area. Therefore, one ***dasht*** (grazing area) can contain more than one community. Generally, these communities have a clear sense of identity (tribally based) and have a clear leadership structure with a community shura. Interviews were conducted at the ‘community-shura’ level.

***Questionnaire development***

The questionnaires were designed by the Assessment Management Team, based upon the information needs and experience gained from previous assessments carried out (including NRVA[[1]](#footnote-2) and REFNAs[[2]](#footnote-3)).

The questionnaires were circulated for comments among the members of the Inter-ministerial Commission on Kuchi, the Kuchi Vulnerability Committee, and the stakeholders. In addition the questionnaire was discussed extensively with the national Kuchi shura to determine the appropriateness and clarity of the questions.

Themes covered in the questionnaire are demographic patterns, actual and preferred migration patterns, integration with settled people, land and house ownership, livestock ownership, water, education, health, labour and income, mines and intervention priorities. The questionnaires are attached in annex III.

***Verification measures***

The assessment methodology is not a census; it relies on reported figures which have an inherent unreliability. A few measures were put in place to reduce the risk for over- (or under) reporting:

- The surveyors were trained to be critical of number of households reported, and were trained to count the tents and/or houses for verification of household numbers. A column was added in the questionnaire for this purpose, which needed to be filled. When comparing the number of tents/houses with the number of households during analysis, a close relationship was found (mean ratio 0.99).
- Pastoralists, with no exception for the Kuchi, are notorious for under-reporting of livestock figures. To remedy this to some extent, the question about livestock ownership was preceded by a question on which vaccines they would require for their livestock. Vaccines are in short supply, and are often demanded by the Kuchi. It was hoped that this question may make them more realistic in reporting their livestock numbers.

***Data collection and management***

Teams were formed consisting of 4 members; one of MRRD, one of MFTA, one of CSO or another member ministry (MoPH, MoE, MAAH, Min. of Mines, MoI) and one hired surveyor. The hired surveyors were experienced interviewers, who were taken from the pool of the most competent NRVA surveyors. In few cases, the national kuchi shura representatives joined the teams.

In February 2004, the teams were sent out to 30 provinces, all except Ghor and Bamyan where no Kuchi stay during the winter. A timeframe of 4 to 6 weeks was allowed for field data collection.

The initial step, at provincial level, was to determine the areas where Kuchi stay. By means of the provincial Kuchi shura these areas were determined and subsequently visited. Often one of the provincial kuchi shura representatives would accompany the team.

Data was collected, screened upon arrival, and entered into a database in Kabul.

***Data analysis and dissemination.***

Due to unforeseen complication in the database development and available analytical expertise, the analysis of the data was delayed for a considerable time.

In the assessment, which was conducted early 2004; the old system of 32 coding provinces and districts based on 32 provinces was used[[3]](#footnote-4).

This report aims at providing a compilation of the information collected, with a general analysis. Where possible, comparisons with NRVA have been made, but caution is required when comparing assessments with different sampling mechanisms.

On the MRRD – VAU website a map has been placed, where basis information on demographic indicators, migration patterns, and use of water sources of all the assessed communities can be accessed directly.

***Validity of the data***

Population figures provided in this report are estimates, and shall be treated as such. There are different reasons for caution:

- Communities may have been missed out, due to inaccessibility, time or motivational constraints of the team to work in difficult areas, bias by the Kuchi leaders, etc.
- In spite of good training of the surveyors and the verification measures put in place, the figures area still are reported figures and are therefore liable to under- or over-reporting.
- Population figures are calculated through multiplying the number of households, which can to some extent be verified, with the average household size which is not verifiable. Small differences in household size can have large implications for population figures.
- Nomadic population are by definition fluid, and individuals, households or communities may change their otherwise predictable pattern for opportunistic reasons. Data collected in one season may change during the next season. However, in general, migration patterns are predictable and community ties are strong which lead to a certain coherence in the community.
- Subjectivity of the questions related to ‘preferred summer area’. Kuchi that have settled may still mention their former summer area as their ‘preferred summer area’, because they would prefer to (still have livestock and) migrate. Others may not. Others may have suffered from disrupted migration patterns over the course of time and may have changed from long-range migratory, via short-range migratory to recently settled. Which area will they call their preferred summer area?

**3. Kuchi categories**

Depending on their actual migration patterns, Kuchi can be divided over three categories; long range migratory, short range migratory, and settled. Long range migratory Kuchi migrate between provinces, short range Kuchi migrate within the province, and settled Kuchi do not migrate at all. According to the definition used in the assessment, all those Kuchi that have settled during the last drought, are still considered Kuchi. Those that settled longer ago are not considered Kuchi in the context of this assessment, even though they may perceive themselves as Kuchi and may still be represented by ‘Kuchi leaders’.

Kuchi that are now settled could have been long-range migratory Kuchi before, or they could have migrated within the province. Equally, those that are currently short-range migratory, may once have been long range migratory Kuchi that lost access to their summer grazing areas. Sub-categories have been formed, to differentiate between those determining factors:

| **Category** | **Sub-category** |
| --- | --- |
| - Long range | - Long range, fully migratory - Long range, partially migratory - Long range, disrupted migration pattern |
| - Short range | - Short range, fully migratory - Short range, partially migratory - Short range, disrupted migration pattern - Formerly long range, now short range, fully migrating - Formerly long range, now short range partially migrating |
| - Settled | - Formerly long range, now settled in winter area - Formerly short range, now settled - Formerly long range, now settled in summer area |

Table 1 Categories of Kuchi

A disrupted migration patterns refers to a situation in which the preferred summer area is not the actual summer area. Chapter 5 provides more background information on the potential reasons behind this disruption.

Partial migration of a community means that a proportion of the community remains behind, while the others migrate to the summer area. This split can even take place within the household; one brother stays and the other goes.

Data has been analyzed on the basis of these categories or sub-categories; where interesting differences appear between the (sub-) categories these are reported.

**4. Demographic patterns**

The Kuchi of Afghanistan, according to the definition used in this survey, number 2.426.304 individuals or 239.859 households.

The long-range migratory Kuchi are predominant (52%), followed by the short-range migratory Kuchi (33%) and lastly the settled Kuchi (15%).

The total number of Kuchi in settled Kuchi communities is 365.106 individuals.

Even in migratory communities, not all households necessarily migrate to the summer areas. In partially migratory communities, some households remain behind and this proportion can vary between 1 and 99%. Detailed information on the level of partial migration is provided in the provinces overviews in annex IV. The total number of non-migrating Kuchi is 967.210 (this number includes settled Kuchi in entirely settled communities and the non-migratory Kuchi in partially migratory communities).

Figure 1 Populations figure per category

The table below shows the overview of Kuchi population figures:

| Total no. of HHs | Total population | No. of HHs not migrating | Population not migrating | Active migrating population | % actively migrating |
| --- | --- | --- | --- | --- | --- |
| **239.859** | **2.426.304** | **93.859** | **967.210** | **1.459.103** | **60.1%** |

Table 2 Overview of Kuchi population figures

An additional 16.056 households were reported as having crossed the border, at the time of the assessment. These were mostly from Nangarhar (7.858 HHs), Khost (6.998), Paktika (661) and Paktya (350). Some caution is required when interpreting these figures, because 1) they were not verified in any way, and 2) entire communities may have been missed out, if they did not have relatives or the tribal liaisons who would have reported these.

***Including those that crossed the borders in winter, the total Kuchi population would be 2.588.719 individuals.***

The map below shows the distribution of the non-migratory Kuchi:

Figure 2 Distribution of non-migratory Kuchi

Kuchi that no longer migrate tend to settle in their winter area, with a few number of exceptions in Ghazni and Paktya where they settled in their summer area. This is probably related to the fact that they own some land in their summer area.

| **Main reason for not migrating** | **relative importance** |
| --- | --- |
| Loss of livestock | 79% |
| Availability of other income in winter area | 9% |
| Security | 6% |
| Became settled | 4% |
| Attitude of resident people in the summer grazing area | 2% |
| Other | 0% |

Table 3 Relative importance of reasons for non-migration

The main reason for not migrating is the loss of their livestock for the majority of the Kuchi. The second reason ‘availability of other income in the area’ signals a more conscious reason to settle, and this likely to be the richer group.

| **Region** | **Loss of livestock** | **Security** | **Became settled** | **Attitude of resident people** | **Availability of other income in winter area** | **Other** |
| --- | --- | --- | --- | --- | --- | --- |
| Central | 82% | 0.5% | 3.7% | 2.3% | 11% | 0.5% |
| East | 71% | 0.9% | 10% |  | 18% |  |
| North | 71% | 20% | 1.4% | 5.6% | 1.8% |  |
| South | 91% |  |  |  | 9.1% |  |
| West | 88% |  | 3.7% | 1.2% | 7.4% |  |

Table 4 Relative importance of reasons for non-migration per region

When broken down per region[[4]](#footnote-5), it transpires that in the north the ‘security’ issue is relatively more important, in the east the ‘availability of other income’ is more important, and in the south the ‘loss of livestock’ is the most important reason. When comparing the main reasons over the Kuchi categories, the obvious result is that ‘loss of livestock’ is the more important reason for the (partially) migratory communities (short-range 92% and long-range 86%), and less important for the settled kuchi (54.2%). For the settled kuchi ‘the availability of other income’ and ‘security’ is relatively more important (both 18%) than for the migratory communities, but still less important than the ‘loss of livestock’.

Due to the migratory patterns of migratory Kuchi, the population figures change per season. The maps below show the population figures per district in the winter and in the summer. Province specific information can be found in annex V, table A.

Figure 3 Population distribution in the winter

Figure 4 Population distribution in the summer

The average household size is 10.1. The household size is slightly larger for the long-range migratory Kuchi than for the other categories. But this household size is significant larger than national average for the rural settled populations (non-Kuchi) of 6.2 per household.

***Migration patterns***

Migration patterns for the migratory Kuchi differ per Kuchi category and per province. Specific information on these migration patterns are provided in the province overviews in annex IV, and in this chapter some rough generalizations are made. For some Kuchi, their preferred migration pattern has been disrupted due to the effects of war and factionalism. For this reason, the Kuchi were asked about their ‘last summer area’ and their ‘preferred summer area’. This chapter deals with the ‘last summer area’ and the next chapter describes the ‘preferred summer areas’.

With a few exceptions, the migration goes from the periphery of the country towards the central areas in the spring season. Summer grazing is predominantly in the foothills of the central highlands and other mountainous regions. However, particularly due to effects of the prolonged war, these disruption patterns have to some extent been eroded. Particularly in the north, large numbers of Kuchi who used to be long-range migratory, and move towards the central highlands now stay within the province and have effectively become short-range migratory. The actual migration patterns of the migratory Kuchi are visually presented in the map on the next page.

Generally, the migration patterns follow the seasons; in spring (Hut or Hamal; February/March) the Kuchi migrate towards their summer areas. The migration period differs, depending on the distance travelled, the climate of that particular year, the amount of pasture on the way and the actual weather. Generally the duration of migration is between 1 and 10 weeks and the return migration is of a similar duration.

The return migration to the winter area usually commences around Mizan or Aqrab (September/October).

In the Northern provinces, from the data it seems that the onset of migration is somewhat later than in the rest of the country. This could be partly caused by spring migration; the Northern provinces tend to use different areas for spring grazing as for summer grazing, which would lead to an initial ‘spring’ migration, followed by a later ‘summer’ migration.

More detailed information on migration patterns can be found in the province overviews in annex IV.

Figure 5 Geographical mapping of migration routes

**5. Pasture**

***Background on access to pasture***

To interpret the results provided in this chapter, it is essential to have some background understanding of the factors that shaped the lack of access to pastures today. This excerpt is taken from the Pastoralist Vulnerability Study, written by the author for WFP[[5]](#footnote-6).

Summer pasture of the majority of the contemporary pastoralists of Afghanistan lies in the Central Highlands. It is not known how frequently pastoralists had used these areas in the past but actions by Abdur Rahman entrenched pastoral access to these areas. He did this by settling Ghilzai Pashtun into the Northern and central areas, as a means of extending his own control into these areas. It was at this time that these central grazing areas were formally opened up to Pashtun pastoralists and the rights of local Hazara to these lands suppressed.

For a period in the 60s and 70s a certain level of symbiosis was present in those areas, with Hazara also benefiting from the presence of the Kuchi as traders and occasionally providers of labour opportunities. Trade on credit basis was introduced into Hazarajat by the Kuchi, and in some cases this led to Kuchi obtaining agricultural land (and the pasture lands associated with it) in exchange for defaulted loans.

Hazara resentment was high. During the Russian occupation period, Hazaras gradually retrieved their lands. During the years of the fight of the mujahedin against the Soviets, and the period of civil war that followed, the grazing areas in the Central Highlands were never fully accessible to the nomads, as they had been before. Migration routes were disrupted due to the chequered control of different commanders controlling different areas and ‘taxing’ the livestock. Many nomads in the north claim to have been looted on numerous occasions, and to have lost their livestock and abandoned the migratory lifestyle in these days, particularly in the north of the country. Local anarchy complicated the necessary migration and created chaos for the nomads as well as for the settled people.

During the Taliban years, the power relations shifted in favour of the Pashtun Kuchi, who to some extent benefited of this situation. The Kuchi are generally perceived to have provided support to the Taliban, which has created tension in the inter-ethnic relations specifically with the Hazaras. Certain groups of nomads were reported as wreaking havoc in the Shomali plains and Ghorband valley by allowing their livestock to graze and destroy vineyards and agricultural lands. Some nomads returned to Hazarajat, where they (had) owned land, and demanded to be paid ‘rent’ for their land, in the shape of harvest produce, which was in some cases taken by force. However, it must be stated that the Taliban did not provide full support to the nomads and tried to control the situation by reducing access of the nomads deep into Hazarajat. According to the nomads, they have not received full access to the Hazarajat since the time of the King (Zahir Shah).

In the present days, the traditional system of pasture rights seems in some cases to be replaced by the power of the gun. Local power relations often determine the rights of access. The payment of taxes to the people who control the pastures (be it villagers, other Kuchi or local commanders) is becoming increasingly prevalent, and the access to pasture is therefore partly determined by poverty levels.

***Access to summer pastures***

A total of 51.485 Kuchi households (524.507 individuals) are currently not migrating to their preferred summer area. This represents 22% of all Kuchi. The districts where these Kuchi would prefer to migrate to are shown in the map below:

Figure 6 District overview of preferred (but not actual) summer areas

The provinces where access to the preferred summer areas is constrained for large numbers of households are notably Saripul, Faryab, Ghor, Wardak and Bamyan.

Saripul province is the preferred summer area for over 12.000 Kuchi households, notably in Kohistanat, Sang Charak and Balkhab districts. These are Kuchi with their winter area in Jawzjan, other districts of Saripul and Balkh province.

Over 6000 households would prefer to migrate to Garzewan, Kohistan and Dawlatabad districts of Faryab province, but currently most of these stay in other districts of Faryab province.

Ghor province (notably Lal Sar Jangal, Chakhcharan and Pasaband district) is the preferred summer area for almost 6000 Kuchi households from predominantly Helmand and Logar provinces, and also Jawzjan, Balkh and Khost provinces.

Wardak province (notably Behsood, Jalrez and Maidan Shar districts) would be the summer area for almost 5000 households, which currently do not have access. These Kuchi come mostly from Nangarhar and Logar and to some extent from Khost and Kabul.

Bamyan (notably Yakawlang and Panjab districts) is the preferred summer area for some 4.500 households mostly from Logar and also from Nangarhar, Balkh, Khost, Samangan and Saripul provinces.

Over 4000 households would prefer to migrate to Parwan province (Panjshir district), from Takhar, Bagram district in Parwan province, and Kapisa.

Ghazni is another province where it is known[[6]](#footnote-7) that access to pasture lands is constrained. According to the data, 1390 kuchi households from Uruzgan, Zabul, Khost and Logar are currently having problems accessing Ajiristan, Malistan and Nawor districts of Ghazni province. From experience gained by the author there should be a large group of Kuchi migrating from Kandahar province to Ghazni in the spring. These kuchi hardly appear in the data at all, and the number of people mentioning Nawor district as their preferred summer area are very limited. This seems to be partly caused by unsatisfactory data collection in Kandahar province, in particular related to the question of ‘preferred summer area’. Another contributing factor will be the fact that this is exactly the group of Kuchi that is currently in the IDP camps of Spin Boldak and Zhare dasht.

The main reasons given for the lack of access to pasture lands are given below.

| **Main reason for lack of access to pasture** | |
| --- | --- |
| Local Commander | 41% |
| Attitude of the resident people | 17% |
| Conversion of pasture land to rain fed land | 13% |
| Usurpation of their land in summer area | 7% |
| Obligatory taxes when using the pasture | 6% |
| Migration route to the area closed | 2% |
| Other | 14% |

Table 5 Relative importance of reasons for lack of access to pasture

The local commander is overall the most important reason for the lack of access to summer pasture. This is followed, with some distance, by the attitude of the resident people.

In the provinces of the north (Saripul, Jawzjan, Bamyan and Ghor for those coming from the north, Balkh, Baghlan, Faryab, and Badghis), the ‘local commander’ is the main reason, followed by ‘conversion of pasture land into agricultural land’ and ‘the attitude of the resident people’ with equal importance. In the south, the ‘conversion of pasture land into agricultural land’ does not appear as such an important reason.

On average 37% of the Kuchi state that they have conflict with the settled people over rights to use the pasture. The graph above shows the distribution over the provinces. The provinces of the north are marked in red, and it can be seen that the problem is indeed quite prevalent in the north.

Figure Percentage of communities that reported conflict with settled people over pasture

Figure 7 Percentage of communities that reported conflict with settled people over pasture

The Kuchi were asked not only about conflict over pasture use, but also if they currently have conflict with settled people on the right to use water sources, to use natural resources, to cultivate rain fed land, and to graze their animals on rain fed belonging to the resident people. Pasture is the main cause of conflict, as can be seen in the graph below.

Figure 8 Percentage of communities reported conflict

Figure 10 Percentage of communities reported conflict

After harvesting, Kuchi often use the rain fed fields for grazing their animals, thereby fertilizing the fields with the manure of the animals. Traditionally, this is a system where both parties benefit from, but conflict may arise, in particular when preconditions for conflict are already present. This is the second reason for conflict, as the graph shows.

Figure 11 Percentage of communities reported conflict

In the winter, the prevalence of conflict is lower, but the reasons are similar as the graph shows.

**Conclusions**

Conflict to summer areas is a major constraint for the Kuchi, leading to 21% of the total Kuchi population not being able to access their preferred summer areas. In addition, a lot of the settled kuchi would have preferred to migrate into these areas as well, but have been forced into becoming settled and change their livelihood pattern. The number of Kuchi suffering from lack of access to summer pastures would be much higher, if these were included as well.

The local commander is perceived to be the greatest threat for the Kuchi trying to access the pasture lands in summer. This leads to some optimism that with decreasing ‘warlordism’ and increasing demobilization this main factor can be removed. The attitude of the resident people will then remain an issue to be dealt with.

It must be understood that under conditions of competition for resources, agriculturalist – pastoralist relations always come under increasing tension, as is known all over the world. Customary arrangements and symbiotic relations carry less weight when neither settled nor nomadic have any surplus for trade or exchange and even livestock for compensation of conflicts is no longer present. However, customary law still exists and it is usually the case that even when under pressure, settled and pastoral people are able to come to working agreements as to how pasture is used. This seems to have been the case during the Zahir Shah period; many informal agreements were developed which allowed seasonal access to pastures, but which were bound by agreement that should livestock interfere with crops, then the pastoralists would pay full compensation.

What these kind of agreements suggest is that there is potential for pastoral and settled people to reconcile their land interests. Local negotiations could be carried out at grass-roots level, to reinstate the customary arrangement and entrench and legitimize these. This process would need to be supported by national and local government, but must have the actual users as the main players in the field[[7]](#footnote-8). In addition, co-existence projects, as those carried out for returnees and returned IDPs in the north, could be implemented in these areas in an attempt to uplift the entire area with benefits for both Kuchi and settled people.

**6. Land ownership**

This chapter provides a general overview of land ownership by Kuchi; province specific information can be found in the province overviews in annex IV. Due to their migratory pattern, Kuchi (short-range and long-range migratory) can potentially own land in their winter location, in their summer location, or in both.

***Proportion of households with land in winter location***

16% percent of the Kuchi own agricultural land in their winter location. The provinces where the largest proportion of Kuchi own land are Paktya, Balkh, Logar and Saripul.

In Paktya up to 96% of the Kuchi households own some land. All of this land is owned by settled kuchi communities; these are ‘former long-range’ and ‘former short-range’ Kuchi. Balkh province (56%), Saripul (52%) and Logar (51%) also have relatively high degrees of land ownership.

In Logar however, 1% is owned by the long-range migratory Kuchi, and none by the short range migratory Kuchi, so almost all of the land is owned by settled kuchi. In Saripul and Balkh the land ownership is more evenly distributed over the Kuchi categories, where some of the long-range migratory, some of the short-range migratory and some of the settled kuchi own land.

Kuchi in Badghis, Ghazni, Helmand, Nimroz, Parwan and Uruzgan own no land at all. Ghazni and Parwan are mostly summer areas, so few Kuchi will stay there during the winter. (Ghor and Bamyan do not have any Kuchi population in winter).

**Figure 12 Distribution of land ownership**

Figure 13 Land ownership per region

A higher proportion of the Kuchi of the central region and the northern region own land, than their counterparts in the other regions[[8]](#footnote-9). In the central region, almost 30% of the communities own land. For more detailed information, please refer to the province overviews.

Figure 14 Land ownership per Kuchi category

The graph shows the percentage of households of the Kuchi of this particular category that own some land. A larger proportion of households in settled Kuchi communities own land, than those in migratory communities. This is according to expectation.

***Amount of land owned in winter location***

On average Kuchi own 1.9 jerib rain fed land and 1.7 jerib of irrigated land per household. When considering only those Kuchi that do own some land (16% of the total Kuchi), the average land ownership per household is 7.6 jerib of rain fed land and 5.1 jerib of irrigated land.

There are in total 100 communities that have relatively large areas of land (> 20 jerib rain fed land), and they drive the average up. The maximum amount of rain fed land owned is 300 jerib per household, in Saripul province. The average land ownership per province can be seen in the graph below.

Figure 15 Provincial overview of average size of land owned

The provinces where the Kuchi have the largest average amounts of land are Kapisa, Saripul, Jawzjan, Baghlan, Paktya, Logar and Balkh. The Kuchi in Saripul and Kapisa own mainly rain fed land, where as those in Jawzjan, Balkh and Paktya own more irrigated land than rain fed land. (refer annex V-B)

Overall, the average number of jerib owned per household is higher in the north than in the other regions. The south scores relatively high on rain fed land, owned by the Kuchi of Kandahar and Paktika. (annex V-B).

Figure 9 Regional overview of average land size

Figure 16 Regional overview of average land size

The settled kuchi own larger areas of land on average than the migratory communities do. The long-range migratory communities own on average less land than the short-range migratory Kuchi, but interestingly they own proportionally a high amount of irrigated land (versus rain fed land). (ref. annex V-B)

86% of the communities state that this land is used for the production of food crops, 10% uses the land for cash crops, and 4% for fodder crops.

Figure 17 Overview average land size per Kuchi category (landowners only)

***Proportion of households with land in summer location***

**Figure 18 Land ownership**

7% of the migratory Kuchi own land in their summer locations. Settled Kuchi that own land in their winter area will still have that same land during the summer. Therefore the settled Kuchi are not taken into consideration. Detailed information on all the provinces is provided in the province overviews. (Annex IV).

A higher proportion of the long-range migratory Kuchi own land in summer than the short-range migratory Kuchi.

The provinces where the largest proportion of Kuchi own land in summer, and their respective winter locations are shown in the table below (in decreasing order of importance):

| **Summer province** | **Winter province** | **No of HHs own land** | **% of HHs own land** | **No of HHs with no access** | **% without access** |
| --- | --- | --- | --- | --- | --- |
| Wardak | Logar | 6110 | 37% | 5210 | 85% |
| Ghazni | Nangarhar, Ghazni, Kandahar | 1130 | 9% | 1100 | 97% |
| Paktika | Paktika | 863 | 14% | 0 | 0% |
| Ghor | Helmand | 592 | 3% | 25 | 4% |
| Herat | Herat | 400 | 3% | 0 | 0% |
| Bamyan | Logar | 380 | 17% | 380 | 100% |

Table 6 Overview land ownership per main summer provinces

The table above shows clearly that access to the agricultural land owned by Kuchi is quite severely compromised, particularly in Wardak, Ghazni, and Bamyan.

The total number of Kuchi households that own land in summer, and can access this land is 2012, which is 0.8% of all Kuchi.

***Amount of land owned in summer location***

Average land ownership in the summer location for those who do have access to the land is 15.8 of jerib of rain fed land, and 1.8 jerib of agricultural land. The average land ownership for those who do not have access to the land is 50 jerib of rain fed land and 92 jerib of agricultural land. This shows clearly that the largest areas of land owned by Kuchi (in the summer location) are in those areas where access is compromised.

91% grows food crops, 4% cash crops and 6% fodder crops.

**Conclusions**

16% of all Kuchi own some land. A higher proportion of the settled Kuchi own land than the migratory Kuchi, and they also own larger tracts of land. But also some of the migratory communities do own land, mostly in their winter location and few in their summer location.

NRVA data show that the average land ownership for the settled people is between 2.7 and 4.4 jerib. This includes both rain fed and irrigated land, either under cultivation or fallow. The average ownership of land for kuchi is 3.6 jerib of land per household (irrigated and rain fed land together). When calculating the average for only those that have some land the average is 7.6 jerib of rain fed and 5.1 jerib of irrigated land (total 12.7 jerib), which is higher than the average land ownership of the settled people!

When comparing with the settled people a different type of picture emerges; high numbers of settled people own small pieces of land, whereas for the kuchi small numbers of people own relatively large tracts of land.

Most land is owned by the Kuchi in the northern and central areas of the country. However, in the central areas, access to this land if often constrained. In Ghazni 97% of the land owned is currently not accessible, in Bamyan 100% and in Logar 85%.

The land owned by Kuchi is mostly used for food crops, so acts in fact as a diversification away from pure pastoralism. Growing livestock fodder can be seen as an additional investment in the livestock economy, to reduce high supplementary feed costs, but growing food crops signals a distinct diversification away from a pure livestock economy.

The Kuchi that own land also possess livestock, in fact the average number of livestock kept per household is higher for the land owners (75 sheep and goats) than the national average for all Kuchi. This is partly caused by the fact that most of the land is owned by Kuchi from the north, where livestock ownership is also higher. However, it signals that Kuchi with land tend to be better-off and more diversified (and there less vulnerable to risk) than the Kuchi without land.

**7. Livestock ownership**

97.7% of the Kuchi assessed own livestock (33 of the communities, in which the poorest households do not own any shoats (sheep and goats)). The distribution of average livestock ownership per household can be seen in the graph. Most households own small numbers of animals.

Sheep and goats are the most important species kept by the Kuchi; and for these two species grouped the term *shoats* is used.

Nationally, the average number of livestock kept per household is 50 sheep and goats (*shoats),* 1.7 camels and 1.2 cows. The settled Kuchi own less shoats than the migratory Kuchi, but the differences in cattle and camel ownership between the Kuchi categories are negligible.

Figure 19 Distribution of livestock ownership

Figure 20 Average livestock ownership per Kuchi category

The average number of shoats (which is the most important livestock species kept by Kuchi) and its distribution over the provinces is shown in the map below (and in annex V-C).

Figure 21 Geographical distribution of livestock ownership (by winter location)

There is a large variation in number of livestock kept per household, and the provinces in the north are clearly much better-off. In Kunduz and Balkh there are communities where the richest households own 1500 shoats, and even the poorest communities own around 100 shoats. Overall, Kunduz and Takhar province stand out very clearly with average number of livestock double those of the other provinces.

Figure 22 Regional[[9]](#footnote-10) overview of average number of livestock kept per household

The average livestock ownership per province and per kuchi category is also presented in the province overviews in annex IV.

Figure 23 Relative importance of livestock diseases

The livestock diseases mentioned by the Kuchi are represented in the graph above. They are presented as an index, since they are composed of the three main diseases mentioned[[10]](#footnote-11).

98% of the Kuchi that have livestock state that they require vaccines; which relative importance is given in the graph below. The vaccines are indexed, because they consist of all three vaccines requested by the Kuchi[[11]](#footnote-12).

Figure 24 Relative importance of vaccines

When available, vaccines can be obtained from Veterinary Field Units. Distance to Veterinary Field Units (VFU) varies over the districts and over the seasons; generally the reported distance is higher in summer than in winter. The average distance to a VFU on foot is 0.6 days in the winter and 0.8 days in the summer. Detailed information per district can be obtained from the Vulnerability Analysis Unit of MRRD, and information at province level is provided in annex V-D.

In a number of provinces, there are some trained Basic Veterinary Workers (BVWs); in Farah, Jawzjan, Kabul, Kandahar, Kapisa, Khost, Kunar and Samangan. On average, for those communities that do have BVWs, they have one BVW per 124 households (range 5-1300).

**Conclusion**

In the current situation in Afghanistan, a lot of Kuchi own small numbers of animals. The average livestock number per household of 50 shoats is relatively low for Kuchi. The estimated minimum number of shoats required for a household to subsist on has been estimated at 100 (for a household of 10)[[12]](#footnote-13). This implies that the average Kuchi in Afghanistan requires obtaining substantial additional income from other sources than the sale of livestock and livestock products.

The provinces in the north are clearly better-off in terms of average number of animals kept. In these areas the average number of shoats kept actually is higher than 100.

Livestock diseases are prevalent and almost all kuchi reported a need for vaccines. Distances to Veterinary Field Units are relatively high, which may be partly caused by the fact that not all existing Veterinary Field Units are actually operational.

Few Basic Veterinary Workers can currently be found within the Kuchi communities. Basic Veterinary Workers could be trained to provide the link between Veterinary Field Units and the Kuchi communities.

**8. Labour and sources of income**

The Kuchi were asked what kind of income generating activities they have, and the overall response was ‘labouring’ or ‘no significant income generating activities. Carpet weaving, transportation services by animals or tractors/trucks, petty trade and masonry was mentioned by very few communities (<5%).

The types of labour opportunities available and how they are distributed over the year can be seen in the graph on the next page.

Harvesting of crops is by far the most important labour opportunity, and over 50% of the Kuchi communities express that they are involved in it. The precise season for harvesting differs over the provinces, but is mostly in Jawza and Seratan which is the wheat harvest. The author has encountered many communities that migrate from one area to the other to benefit from the labour opportunities created by harvesting in two provinces[[13]](#footnote-14).

Planting of crops is the next important source of labour, mostly in Aqrab and Mizan (Sept/Oct), followed by ‘construction’. The latter is more evenly distributed over the year, with peaks in Hamal, Saur, Jawza (March-May) and Mizan and Aqrab (Sept-Oct).

Shepherding, the herding of animals for another person in return for a salary or a fixed proportion of the livestock products (including off-spring), is another relatively important source of income throughout the year[[14]](#footnote-15). Collection of firewood is another relatively important labour opportunity that Kuchi use.

Harvesting, planting of crops and other farm work are important labour opportunities, but they are of limited duration (one or maximum two months). Labour opportunities that can provide some income throughout the year are ‘construction’ and ‘shepherding’, and also weaving, barter and trade, and firewood collection.

As can be seen above, Kuchi are mostly involved in unskilled labour. They were asked which vocational skills training they would be interested in. Women would be interested in handicrafts, like carpet weaving, tailoring and embroidery. Men are mostly interested in livestock production skills. This is interesting, since it implies that they feel there is more to learn in the field they are already engaged in. On the other hand, it may also signal an absolute preference for livestock keeping, and lack of interest in learning new skills.

Figure 25 Availability of labour opportunities per month

**Conclusions:**

Availability of labour opportunities is limited, and the most important labour opportunities that Kuchi are involved in are of short duration. Particularly the poorer Kuchi rely to a large extent on additional income sources. Mostly the Kuchi are involved in unskilled labour (with the exception of shepherding), and they would be interested in learning new skills.

Vocational training programs could be implemented for the Kuchi, in particular for those who have few or no livestock. It is known[[15]](#footnote-16) that often Kuchi households have one or more members working as migrant labourers in main bazaars, in an attempt to support the household. This could be an important target group for vocational training programs.

**9. Water**

**Water for human consumption**

***Types of water sources used***

The relative use of different water sources used by the Kuchi in their winter areas is presented in the graph below[[16]](#footnote-17).

The open well is clearly the most important source of water for the Kuchi, followed by the use of water from the rivers and springs. Water from all sources is generally considered unsafe, with between 84% and 99% of the interviewed stating it is unsafe. The water from the spring is perceived as being slightly more safe (16% considers it safe), as well as hand pump (10%) and the open well (9%).

Figure 26 Relative importance of type of water source for human consumption (in winter)

Considering the fact that water from a hand pump should generally be safe water, the Kuchi seem to have a very negative perception of the quality of the water, even when this does not seem justified. Interestingly however, they have more trust in springs than in hand pumps.

If one considers all types of water sources other than hand pumps to be unsafe water, then 90% of the Kuchi do not have access to safe water.

The graph below shows the use of water sources of human consumption in the summer locationsError: Reference source not found. In summer, springs really gain in importance as well as the rivers, particularly for the migratory Kuchi. Open wells and hand pumps are used rarely, and then mostly by the settled kuchi (who would generally be in their winter area). The summer areas of the Kuchi are much more remote and are often in the mountains where there are large grazing areas but no settlements and no water sources of the settled people that they can use. In these areas they are therefore mostly dependent on natural water sources.

Figure 27 Relative importance of type of water source for human consumption (in summer)

***Potential conflict over water sources***

In the winter, 34% of the communities state that they stay far away from the settled people, whereas in summer this increases to 67%. Of course, this difference stems from the migratory communities, and when considering only the migratory Kuchi this figures increases from 71% stays far from the settled people, and their water sources, in summer.

This is also substantiated by the fact that in winter the Kuchi tend to share the water sources more with the settled people than they do in summer. For the settled kuchi there is of course no real difference, but for the migratory communities there is; in winter 77% shares their water sources with the settled people, and in summer only 54%.

Slightly more conflict over water sources is also reported in the summer areas than in the winter areas. The long-range migratory Kuchi report the highest levels of conflict over water sources (21%).

***Distance to water***

Nationally, the average distance to the most important water source is 0.8 hours in winter and 1.0 hours in summer. The distance to water varies greatly over the districts, and is represented in the map below. (refer annex V-E for province specific information).

On the MRRD – VAU website, information is provided on the most important water source and the distance to the most important water source for every community.

Figure 28 Distance to most importance water source in hours (in winter)

Figure 29 Distance to most importance water source in hours (in summer)

**Water for livestock**

The sources of water used for the livestock are presented in the graph below.

Figure 30 Relative importance of type of water source for livestock (in winter)

The river and the open well are the most important sources for the livestock in the winter. When compared to the water sources used for human consumption, one can notice that they tend to use the rivers more for livestock and the open wells more for human consumption. This must be related to the quantity of water required for livestock, which rivers can provide more easily.

The graph below shows the water sources used for livestock in the summer. In the summer, springs are important sources of water, as well as the rivers. There is no obvious difference between the water sources used for humans and for livestock in the summer. This must be related to the lack of other sources, animals and people have to use the same sources for lack of alternatives.

Figure 31 Relative importance of type of water source for livestock (in summer)

**Conclusions**

90% of the Kuchi don’t have access to safe water, which is a very high figure. As a comparison; 76% of the settled people do not have access to safe water.

There is obviously a need for more safe water sources (e.g. hand pumps) for human consumption. Livestock will need to continue to use the natural sources, to avoid extracting large quantities of water from the ground water. In those areas where natural sources are not available water pans could be dug for the livestock.

The specific areas where water development is most required can be obtained from the Kuchi-maps posted on the MRRD-VAU website.

**10. Health care**

***Involvement in immunization campaigns***

The level of involvement of Kuchi in national immunization campaigns was assessed through determining the percentage of children vaccinated for Polio, DTP, Measles, and having received Vit. A. To remind the people, Polio was described as ‘vaccination drops in the mouth’, DTP as ‘injection in the thigh’, Measles as ‘injection in the upper arm’, and Vitamin A as ‘capsules in the mouth’.

Vaccination rates of children vary considerably between the provinces, as can be seen in annex V-F. Lowest vaccination rates can be found in Laghman, Uruzgan, Zabul, Helmand, Samangan and Paktika. On average, the vaccination rates in these provinces are below 25%, with the exception of the Polio vaccination which reaches up to 34-39% in Helmand and Paktika. Overall, it transpires that the vaccination rates for Polio and Vitamin A are slightly higher than those for Measles and DTP. The reasons for this difference could be various, and the data would need to be compared with the MICS survey and micronutrient survey results for some potential answers.

The provinces with the highest vaccination rates are Farah and Faryab with 100% for all 4 types, but also Kunar, Paktya, Takhar and Wardak scored high, with vaccination rates over 85% for all, with the exception of Takhar which had a DTP vaccination rate of 67%.

The migratory factor, which could be expected to be an important determining factor for level of involvement in immunization campaigns, does not come out clearly. In fact, the highest percentages are found in the settled kuchi populations (as might be expected), but this is followed by the long range migratory groups which is unexpected.

***Use of health facilities***

The level of access to different health facilities was assessed, through determining the type of health facility used (‘health centre/mobile outreach clinic’, pharmacy/drug seller’, ‘hospital’, ‘traditional healers’, ‘traditional birth attendant’, ‘religious healer/shrine’, and ‘private doctor’), the distance to these facilities, who can access them, whether they trust them and for which reason.

The health facility most used by men is the private doctor, followed by the hospital and the religious healer or shrine. Women also use the private doctor most, but for women the traditional birth attendant is of almost equal importance. Overall use of health facilities is slightly higher in winter than in summer, but the type of facility used is the same. There is no apparent difference in types of health facility used by the long-range, short-range or migratory Kuchi. Information about health facilities used per province is provided in annex V-G.

The overall usage of health facilities reported is highest in Wardak, Faryab and Baghlan. This is most likely more related to the way of questioning of the interviewers, than to access of the kuchi to these health facilities.

Figure 32 Relative importance of various health facilities

For those Kuchi that use the above facilities, the Kuchi report that everyone has access to these facilities, men and women both. Only for the hospitals it was mentioned in 6% of the cases that only the men have access, and traditional birth attendants are logically only used by women.

Trust levels for these facilities are quite low. The graph below shows the levels of trust amongst those that use these facilities (‘users’) and all Kuchi (‘all’). It transpires that even among those that use the facilities, and who one would expect have some level of trust in these facilities, trust levels are low.

Figure 33 Percentage of communities which expressed trust in the health facility

People have the most trust in religious healers/shrines (91% of users), followed by traditional birth attendants (46% of users), the hospital (44% of users) and the private doctor (42% of users).

The main reasons for this lack of trust differ for the different health facilities are given in table 6:

| **Health facility** | **First reason** | **Second reason** |
| --- | --- | --- |
| Health centre | lack of medicine | too far |
| Pharmacy | too expensive |  |
| Hospital | lack of medicine | too far |
| Traditional healer | Poor quality doctor |  |
| Traditional birth attendant | Poor quality doctor |  |
| Private doctor | Too expensive |  |

Table 7 Reasons for lack of trust in health facility

Interestingly, the answer ‘discrimination against Kuchi’ was only given in 6% of the cases, and against expectation even less in the summer areas.

***Distance to health facilities***

Traditional birth attendants, traditional healers and religious healers are mostly reported to be in the community, which considerably reduces the average time travelled. Hospitals, pharmacies, health centres and private doctors are mostly located in towns, which require further travelling. In the summer time the Kuchi stay in more remote areas and travel times increase, as can be seen in the graph below.

Figure 34 Average distance to health facility on foot

In the winter areas, the districts in which the longest distances (over one day) to a basic health centre were recorded are: Kishim, Tagab and Tashkan in Badakhshan province, Baghlan Jaded in Baghlan province, Adraskan in Herat province, Surobi in Kabul province, Kharwar in Logar province, and Zurmat in Paktya province. When considering also the summer areas, the following districts can be added to the list; Jurm in Badakhshan, Tolak in Ghor province, Farsi in Herat district, Deh Sabz and Paghman in Kabul province, Panjshir in Parwan province, and Jalrez and Neerkh in Wardak province.

92% of the men, and 76% of the women would be interested in being trained as Basis Health Worker; and 100% of the women would like to be trained as Birth Attendants.

**Conclusion**

The Kuchi seem the have the highest level of trust in the religious leaders and shrines, which they also tend to use quite often. However, the traditional birth attendant, the private doctor and the hospital are also used relatively often, even though trust levels are lower.

Improving access to health care would need to include a component of awareness creation on the effectiveness of modern medicine when used in the proper manner. A shift away from religious healers, towards the use of more modern health care could be furthered through training of community based health workers. These could provide mid-wife and basic health care services within the community, but could also serve as a link to health care centres in the vicinity.

Health centres, targeted at the Kuchi communities, need to be established in the more remote areas, in particular in the summer areas. Vaccination campaigns need to be planned together with the Kuchi to ensure their presence at the time of the vaccination. This document can act as a tool for planning.

**11. Education**

Literacy levels, e.g. the number of men and women that can read and write in the community are very low; on average 2.0% for men and 0.05% for women. Literacy rates are calculated as the number of individuals who can read and write as a percentage of the total number of individuals aged over 15 yrs[[17]](#footnote-18). (refer to annex V-H for a province overview)

The highest level of male literacy was found in Baghlan (6.6%), Balkh (6.1%), Logar (5.6%) and Badakhshan (5.1%), and for women in Saripul (0.4%) and Baghlan (0.3%).

Literacy levels seem higher among the settled kuchi, and lowest among the short range migratory Kuchi (refer annex V-I). This is surprising, considering that access to education must be even more difficult for the long-range migratory Kuchi. This effect seems to be caused to some extent by a small group of ‘disrupted long-range migratory Kuchi’, which has a relatively high literacy rate (1.9%). This may not be a significant difference however.

Figure 35 School attendance rates for boys in the winter location.

The national average of Kuchi children attending school is 6.6% for the boys and 1.8% for the girls. The graph above shows the distribution over the provinces for the boys. (refer annex V-H)

In Balkh province, 47% of the boys and 25% of the girls go to school during the winter, which is the highest among all the provinces. In the summer these figures drop to 1.7% and 0.4% as national average, and to 25% and 20% in Balkh province. In general the northern provinces of Badakhshan, Baghlan, Kunduz, Samangan, and Saripul score relatively high, as well as Logar, Nuristan and Wardak.

The average walking time to primary school in the winter area ranges from 0 to almost 5 hours, with a national average of 1.9 hours. In the summer area, average walking time is considerably higher, with a national average of 2.6 hours (range 0-12(!) hours). The walking distance to schools in general is somewhat lower for the settled Kuchi, and longer for the short-range and long-range migratory Kuchi.

Figure 36 Average walking distance to primary school

The highest average walking times were recorded in Herat, Jawzjan, Ghazni and Kunduz provinces. Distances over 5 hours to the primary schools for boys were recorded in Sholgara of Balkh province, Muqur of Ghazni, Adraskan, Obeh, Pashtun Zarghoon and Shindand of Herat, Qoshtepa of Jawzjan, Najrab and Tagab of Kapisa, Behsud of Nangarhar, and Sayad of Saripul province. In the summer provinces, some of the districts above re-appear, and the following districts can be added to the list: Dolina, Shahrak and Tolak of Ghor province, Chest-e-sharif, Farsi, Karukh, and Kushk of Herat province, Pul-e-alam of Logar, and Panjshir of Parwan.

The longest walking time recorded for the winter time is 12 hours recorded in Shindand district of Herat province. The longest walking time recorded in a community was 35 hours (!) in Tolak district of Ghor province both in the summer. These numbers may be over-reported since other communities from the same area in winter (Pashtun Zarghun district in Herat province) also report long distances for those that stay in Pashtun Zarghun district in winter. This could be correct, since these are short-range migratory, but it is suspicious. In communities with such long walking distances, the children are in fact not going to school.

The main reasons given for low school attendance for boys are ‘schools are not available’(44%) and ‘schools are far away’ (32%). For girls the main reasons given are also ‘schools are not available’ (58%), followed by ‘tradition’ (18%), and ‘too far away’ (15%).

In comparison, according to NRVA, for the settled people 30% of the boys go to school and 15% of the girls. The unavailability of schools is the main reason for the settled people as well, but only reported in 20% of the cases, which is far lower than for the Kuchi.

Figure 37 Relation walking time and school attendance

The graph shows the relation between walking time (to primary school in winter) and attendance levels (proportion of the boys going to school in winter). As walking distance increases, the attendance decreases. There is a significant inverse correlation between walking time to school and proportion of boys going to school (P<.001, R^2^=0.079)

76% claimed that they would send their boys to a boarding school, and 24% would send their boys to a day school. Most likely, this response reflects the reality on ground, where day schools are too far away. Boarding schools will therefore be perceived as the only option by many Kuchi. (Kuchi are used to the concept of boarding schools as some of these were established in the 1970s). If schools were available at a closer distance or in the community, they might still prefer day schools.

For the girls, it is a different story; only 1% would be allowed to go to boarding school, whereas 69% would agree to send their girls to a day-school. This is clearly a question of mentality, where they would not allow their girls to stay in a hostel.

Detailed information is available on the districts where the Kuchi would prefer to have the schools built, and can be obtained from the Vulnerability Analysis Unit of MRRD.

The Kuchi were also asked if there were individuals available in the community that could be trained in a 3-month program, to become community based teachers. This question was asked to gain an understanding if the Kuchi believe that they would be able to build the capacity to teach their own children. A 3-month program was chosen to encourage the people to select those individuals who already have some skills.

Indeed, a significant correlation can be found between the number of men that can read and write and the number of men that can be trained, as the graph shows.

**Figure 38 Relationship literacy and no of men can be trained**

The percentage of communities that would have some individuals that could be trained and work as a community-based teacher is presented in the graph below.

Figure 39 Percentage of communities with at least one member to be trained as ‘community teacher’

The variation between the provinces is quite high. Particularly noticeable are the provinces of Badghis, Badakhshan, Zabul, Khost, Helmand, Khost and Uruzgan where none or very few of the communities reported to have such individuals.

**Conclusions:**

Literacy rates are extremely low, and even the school attendance rates for boys and girls are low which does not signal a change in this situation. The main reason for low school attendance rates is the ‘lack of schools’ and it is therefore not a question of attitude or lack of willingness (at least not for the boys).

Considering the fact that many Kuchi stress the importance of education and learning new skills[[18]](#footnote-19), the current situation needs to be reversed.

An education strategy needs to be developed, which could include a combination of fixed schools with a mobile extension. Particularly in the remote summer areas of Herat and Ghor, schools are not available at all, and need to be constructed. However, this should be done in the context of a larger strategy for improving access to education for Kuchi.

Literacy training for adults should also be considered alongside education of the children.

**12. Mines**

The Kuchi were asked if there are any land mines in their areas, both their summer areas and their winter areas. The map below shows the reported presence of mines in the Kuchi areas.

Figure 40 Reported presence of mines

The presence of mines is reported as highest in Baghlan, Nuristan and Kabul. Overall, 9% of the Kuchi state there are mines in their summer locations, and 10% state there are mines in their winter location.

Interestingly, there is no obvious relation between the Kuchi category and the extent they are affected by presence of mines. In fact, a slightly larger proportion of the settled kuchi are affected by mines than of the migratory Kuchi. This can be partially explained by the fact that many settled Kuchi have settled in the same area as where they were staying before, and are therefore still at equal risk of being affected by mines.

It is mostly the access to pasture land that is affected; for the settled and the migratory kuchi communities, both in summer and winter areas. This is followed at some distance by the access to agricultural land. The migratory routes are mentioned only in few cases, and mostly by the long-range migratory Kuchi.

**Conclusions**

Demining exercises should include the Kuchi areas, upon guidance of the Kuchi themselves.

**13 Intervention priorities**

The Kuchi were asked what prioritized needs they would like the Afghan government to address. Up to three interventions could be given, and the table below reflects the first priority given.

| **Description of intervention priority** | **Relative importance** |
| --- | --- |
| Improved drinking water quality/quantity | 29% |
| Rehabilitation of irrigation system | 2% |
| Construction or repairing of rural road | 1% |
| Construction of new roads to improve rural access | 0% |
| Improvement of health care in the area | 8% |
| Increased of education facilities in the area | 3% |
| Improvement in housing or provision of shelter | 11% |
| Improved veterinary services | 2% |
| Micro-credit schemes | 4% |
| Employment opportunities | 4% |
| Literacy training | 0% |
| Vocational skills training | 2% |
| Restocking | 23% |
| Food assistance | 7% |
| De mining of the area | 2% |
| Other | 15% |

Table 8 Frequency of the first intervention priority

When taking into account the other two priorities that they were asked to mention, it transpires that the following five interventions are considered the most important; 1) drinking water, 2) restocking, 3) improved health care, 4) shelter / housing, and 5) increased education facilities.

The relative importance of each of these 5 interventions in the provinces is presented in figure 41 on the next page.

Interestingly, those that mentioned ‘restocking’ as their most preferred intervention are not necessarily those with less animals. The average number of shoats kept by those that want restocking is close to the national average (50 shoats). In fact, even those that own on average more than 100 shoats, still mention ‘restocking’ as their main preferred intervention! This may partly be caused by a preference for livestock keeping over other activities, which is then reflected in an answer such as this.

**Conclusions**

Drinking water, health care and education are primary needs, which the Kuchi hope the government will be able to fulfil for them. There is a surprisingly high demand for ‘restocking’, even among those that do have relatively large herds.

Figure 41 Provincial overview of intervention priorities

**14 Conclusions and recommendations**

The total number of kuchi included in this assessment is ***2.426.304 kuchi*** (or 2.588.719 when including those that had crossed over the border at the time of the assessment).

Depending on migration patterns, Kuchi can be divided into three main ***categories***; 1) long-range migratory (migrating from province to province), short-range migratory (migrating within the province), and the recently settled (that settled due to the effects of the last drought). The long-range migratory Kuchi are predominant, followed by short-range migratory Kuchi, and lastly the settled.

Some communities are partially migrating, with some households remaining behind in the winter area when the others migrate to the summer areas. In total almost 40% of the kuchi are currently ***not migratory***. The main reasons are ‘loss of livestock’ and particularly in the north security and the conversion of the pasture land into agricultural land are other contributing factors.

22% of the kuchi state that they are currently not migrating to their preferred summer areas; particularly those areas situated in Saripul, Faryab, Ghor, Wardak and Bamyan. Some of these migrate to other areas instead or have stopped migrating all together. Local commanders are the main hazard, followed by the attitude of the resident people.

Due to the fact that the summer pastures are essential for livestock grazing, this ***lack of access to pasture*** has serious negative effects on kuchi productivity, and is therefore of pivotal importance and needs to be addressed urgently. Grass-roots level pasture negotiations[[19]](#footnote-20) can revive past customary arrangements and conflict resolution mechanisms, and entrench and legitimize these. The presence and support of central and provincial government will be essential. Preferably, this approach would be supported by a technical range land assessment which will provide information on the status of the range land and potential recommendations for improved range land management.

16% of the Kuchi ***own land***, and the average land ownership for these kuchi is higher than amongst the settled people. In addition, the kuchi land owners also own livestock, and on average even more than the non-land owning Kuchi. The land owners are therefore clearly better off, both in terms of asset ownership as in terms of increased diversification leading to decreased vulnerability. The highest percentage of land ownership and the largest tracts of land owned are in the northern and central provinces. Most of this land is owned in the winter location; when land is owned in the summer location the access to this land is often constrained, particularly in Wardak, Ghazni and Bamyan.

98% of the Kuchi own ***livestock***, and on average they own 50 shoats (sheep and goats), 1.7 camels and 1.2 cows. The average livestock number per household of 50 shoats is relatively low for Kuchi. The estimated minimum number of shoats required for a household to subsist on has been estimated at 100 (for a household of 10)[[20]](#footnote-21). This implies that the average Kuchi in Afghanistan requires obtaining substantial additional income from other sources than the sale of livestock and livestock products. Restocking is the second preferred intervention by the Kuchi, and interestingly so not only for those with very low numbers of livestock, but also those with average numbers of livestock. This shows that indeed the current livestock numbers are too low for a sustainable livelihood.

***Labour opportunities*** are limited, and generally of limited duration and highly seasonal. Due to the lack of skills, most Kuchi are involved in unskilled labour. Vocational training programs to boost the skill levels of the kuchi would greatly contribute to reduced vulnerability. Even migratory households, and in particular the poorest, often have one or two household members working as casual labourer in a market and increasing their skill levels would assist the entire household.

Only 10% of the Kuchi have access to ***safe water***; they rely heavily on open wells, springs, and rivers. The hand pump is only the fifth important source of water, which is the only source of water considered safe according to UNICEF’s standards. Often the Kuchi use the water sources of the settled, which can in cases lead to conflict. There is a clear need for development of more safe water sources, particularly in the more remote areas.

Lack of drinking water is the most preferred intervention for the Kuchi.

***Access to services***

Access to ***education*** is limited, with extremely low literacy rates (2.0% of men and 0.05% for women). School attendance rates are also low which does not signal a change in this situation.

Providing recommendation on how to develop an education strategy for Kuchi goes beyond the scope of this report, but a few remarks can be made. The lack of schools or long distance to schools in the impeding factor in school attendance. At least for the boys, there seems to be a positive attitude towards education. The migration patterns cause the Kuchi to stay for relatively long period (of around 5 months) in one place, at which times education could be provided in those areas. Either a adaptation of the school year, so that the school holidays coincide with the migration period, or a mobile arm connected to the school during the periods that the kuchi are migrating or scattered and far away in the mountains.

Considering the low literacy rates among the adults, the availability of teachers from kuchi background willing to teach the Kuchi in their areas will be a major constraint. Local people who received a basis training (‘barefoot teachers’) could possibly play a role, if not in actually teaching, then possibly in maintaining some supervision of the students during the times that education is not on-going.

Access to ***health care*** seems to be constrained by two main factors; 1) the lack of health facilities in the areas where Kuchi are, and 2) the lack of trust in these facilities. Any approach to health care would need to address these two issues.

Training of community based health workers could contribute to these objectives, through providing basic health care directly to the community, through creating a link to the existing health facilities in the area, and can act as a vehicle for community awareness on the use and efficacy of modern medicine. This will need to be supported to some extent by the establishment of health facilities for the Kuchi, particularly in the remote areas where there are no existing health facilities.

***Veterinary services*** are important for Kuchi; a clear example is the fact that 98% of the kuchi state that they need vaccine for their livestock. The availability of vaccine is actually one of the main constraints[[21]](#footnote-22). For the privatized Veterinary Field Units, the Kuchi are actually their potentially best source of income, since they are willing to invest in veterinary care and have a lot of animals to invest in[[22]](#footnote-23). Therefore they are an important target market for the VFUs, which has not been sufficiently tapped into yet.

The training of Basic Veterinary Workers, which has been done to some extent in the past as can be seen from the few remaining Basic Veterinary Workers in some communities, can bring the veterinary services closer to the Kuchi communities, and can improve joint planning of vaccination campaigns and supply of other essential medicines between the VFU and the kuchi.

**Annex I: Translation of months**

In the text the Persian words for the months are often used, and they translate as follows:

| **Month** | **From - to** |
| --- | --- |
| Hamal | 21^st^ March – 20^th^ April |
| Saur | 21^st^ April – 20^th^ May |
| Jawza | 21^st^ May – 20^th^ June |
| Seratan | 21^st^ June – 20^th^ July |
| Asad | 21^st^ July – 20^th^ August |
| Sombola | 21st August – 20^th^ September |
| Mizan | 21st September – 20^th^ October |
| Aqrab | 21st October – 20^th^ November |
| Qaus | 21st November – 20^th^ December |
| Jedi | 21^st^ December – 20^th^ January |
| Delwa | 21^st^ January – 20^th^ February |
| Hut | 21^st^ Feb – 20^th^ March |

**Annex II: Regional Categories of Provinces**

| **Region** | **Provinces** |
| --- | --- |
| North | Badakhshan, Balkh, Faryab, Baghlan, Jawzjan, Kunduz, Samangan, Saripul, Takhar. |
| East | Khost, Laghman, Nangarhar, Kunar, Nuristan. |
| Central | Ghazni, Kabul, Kapisa, Logar, Paktya, Parwan, Wardak, Bamyan, Ghor. |
| South | Helmand, Kandahar, Nimroz, Paktika, Uruzgan, Zabul. |
| West | Badghis, Farah, Herat. |

**Annex III, IV, V**

To be provided upon request

1. National Risk and Vulnerability Analysis [↑](#footnote-ref-2)
2. Rapid Emergency Food Needs Assessments [↑](#footnote-ref-3)
3. The only exceptions are the districts of Azra and Kahmard which in the old system belong to respectively Paktya and Baghlan, but for the purpose of this assessment have been placed in respective provinces of Logar and Bamyan to which they now belong. [↑](#footnote-ref-4)
4. Refer to annex V for the distribution of the provinces over the regions. [↑](#footnote-ref-5)
5. Source: Pastoralist Vulnerability Study, 2002, by Frauke de Weijer, for WFP VAM-Unit. [↑](#footnote-ref-6)
6. through the long involvement of the author with the Kuchi and Afghanistan. [↑](#footnote-ref-7)
7. Refer Liz Alden Wily, ‘Peace on the Pastures’, 2004, AREU publication, www.areu.org.af [↑](#footnote-ref-8)
8. Refer to annex V for the distribution of the provinces over the regions. [↑](#footnote-ref-9)
9. Refer to annex V for the distribution of the provinces over the regions. [↑](#footnote-ref-10)
10. Index = (1^st^ disease mentioned (%) * 3 + 2^nd^ mentioned (%) * 2 + 3^rd^ mentioned (%) * 1) / 6) [↑](#footnote-ref-11)
11. Index = (1^st^ vaccine requested (%) * 3 + 2^nd^ vaccine requested (%) * 2 + 3^rd^ vaccine requested (%) * 1) / 6) [↑](#footnote-ref-12)
12. From interviews held by the author over the years, and also substantiated by B. Glatzer who states the minimum number of sheep is 10 per adult member of the household. (*Glatzer and Casimir,* Herds and households among Pashtun pastoral nomads; limits of growth*, Etnology, Vol. XXII. No. 4, 1983)* [↑](#footnote-ref-13)
13. Source: Microfinance for Kuchi, background information and recommendations, by Frauke de Weijer, for MISFA. [↑](#footnote-ref-14)
14. For more information on shepherding contracts, refer to ‘*Microfinance for Kuchi, background information and recommendations*, 2005, by Frauke de Weijer, for MISFA. [↑](#footnote-ref-15)
15. Refer to ‘*Microfinance for Kuchi, background information and recommendations,* 2005, by Frauke de Weijer, for MISFA. [↑](#footnote-ref-16)
16. This distribution is based upon all sources (with a maximum of three) mentioned by the Kuchi as being important. [↑](#footnote-ref-17)
17. The typical demographic age distribution for developing countries was used as the basis for calculation (*Food & Nutrition Needs in Emergencies, (UNHRC, UNICEF, WHO WFP*)  The number of men aged over 15 yrs was estimated at 48.52% of total men and for women at 49.36% of total women. [↑](#footnote-ref-18)
18. From personal experience gained through author’s long involvement with Kuchi [↑](#footnote-ref-19)
19. For more information on the suggested approach, refer to Liz Alden Wily; peace on the pastures, 2004. www.areu.org.af. [↑](#footnote-ref-20)
20. From interviews held by the author over the years, and also substantiated by B. Glatzer and F. Casimir who state the minimum number of sheep is 10 per adult member of the household. (*Glatzer and Casimir,* Herds and households among Pashtun pastoral nomads; limits of growth*, Etnology, Vol. XXII. No. 4, 1983*) [↑](#footnote-ref-21)
21. Source; Anne Lancelot, Madera Country manager, personal communication. [↑](#footnote-ref-22)
22. Source; Abdul Halim, Head of Veterinary Sector, Madera Laghman field office. [↑](#footnote-ref-23)
